# A Small Peptide Increases Drug Delivery in Human Melanoma Cells

**DOI:** 10.3390/pharmaceutics14051036

**Published:** 2022-05-11

**Authors:** Shirley Tong, Shaban Darwish, Hanieh Hossein Nejad Ariani, Kate Alison Lozada, David Salehi, Maris A. Cinelli, Richard B. Silverman, Kamaljit Kaur, Sun Yang

**Affiliations:** 1 Department of Pharmacy Practice, Chapman University School of Pharmacy, Irvine, CA 92618, USA; fong149@mail.chapman.edu (S.T.); klozada@chapman.edu (K.A.L.); 2 Department of Biomedical and Pharmaceutical Sciences, Chapman University School of Pharmacy, Irvine, CA 92618, USA; shaban_darwish@yahoo.com.sg (S.D.); haniehariani@gmail.com (H.H.N.A.); dsalehi@chapman.edu (D.S.); 3 Center for Developmental Therapeutics, Department of Chemistry, Department of Molecular Biosciences, Chemistry of Life Processes Institute, Northwestern University, Evanston, IL 60208, USA; macinell@nmu.edu (M.A.C.); r-silverman@northwestern.edu (R.B.S.); 4 Department of Pharmacology, Feinberg School of Medicine, Northwestern University, Chicago, IL 60611, USA

**Keywords:** peptide, melanoma, uptake, tumor xenograft, targeted delivery, nNOS inhibitors

## Abstract

Melanoma is the most fatal type of skin cancer and is notoriously resistant to chemotherapies. The response of melanoma to current treatments is difficult to predict. To combat these challenges, in this study, we utilize a small peptide to increase drug delivery to melanoma cells. A peptide library array was designed and screened using a peptide array-whole cell binding assay, which identified KK-11 as a novel human melanoma-targeting peptide. The peptide and its D-amino acid substituted analogue (VPWxEPAYQrFL or D-aa KK-11) were synthesized via a solid-phase strategy. Further studies using FITC-labeled KK-11 demonstrated dose-dependent uptake in human melanoma cells. D-aa KK-11 significantly increased the stability of the peptide, with 45.3% remaining detectable after 24 h with human serum incubation. Co-treatment of KK-11 with doxorubicin was found to significantly enhance the cytotoxicity of doxorubicin compared to doxorubicin alone, or sequential KK-11 and doxorubicin treatment. In vivo and ex vivo imaging revealed that D-aa KK-11 distributed to xenografted A375 melanoma tumors as early as 5 min and persisted up to 24 h post tail vein injection. When co-administered, D-aa KK-11 significantly enhanced the anti-tumor activity of a novel nNOS inhibitor (MAC-3-190) in an A375 human melanoma xenograft mouse model compared to MAC-3-190 treatment alone. No apparent systemic toxicities were observed. Taken together, these results suggest that KK-11 may be a promising human melanoma-targeted delivery vector for anti-melanoma cargo.

## 1. Introduction

Human cutaneous melanoma (CM) is one of the few cancers in which the incidence rate continues to increase, thereby making this disease a rising public health concern in the United States. The advances in the field of molecularly targeted therapeutics and immunotherapy have changed the landscape of melanoma management and significantly improved patient survival [1]. However, the quick development of drug resistance to targeted therapy, and the fact that only a subset of melanoma patients respond to immunotherapy [2], have urged researchers to continuously investigate new and improved strategies for anti-melanoma treatment [3].

Compared to many human malignancies, CM is highly resistant to traditional cytotoxic chemotherapy. The only FDA-approved cytotoxic drugs for melanoma treatment are dacarbazine and temozolomide, which have very limited efficacy. Although doxorubicin (DOX) is highly effective in the treatment of many types of cancer, melanoma is resistant to its cytotoxic effects as a result of the intrinsic resistance of this cancer type to DOX [4,5,6]. Similar resistance was also reported with cisplatin treatment in melanoma [7]. An enzyme, neuronal nitric oxide synthase (nNOS), was found to be overexpressed in CM, and has been identified as a key player in melanomagenesis by enhancing tumor growth and interferon-gamma-stimulated melanoma progression [8,9]. Our previous studies demonstrated that novel nNOS inhibitors, such as MAC-3-190 and HH044, exhibit promising anti-melanoma activities by inhibiting nNOS-mediated nitric oxide signaling [8,9,10,11]. 

The mechanism of drug resistance in melanoma is complex [12,13]. Increased drug efflux is one of the most observed mechanisms, resulting in reduced intracellular drug levels suboptimal for cytotoxicity [14]. A practical approach to overcoming drug resistance is to improve drug delivery and, as a result, increase intracellular drug accumulation and efficacy [13]. Different strategies are used to increase the uptake of drugs preventing drug efflux, such as the use of drug carriers like liposomes or nanoparticles [15], drug conjugates where the drug is covalently conjugated to a targeting ligand (e.g., peptides) [16,17,18], and co-administration of drugs with targeting ligands [19]. While drug carriers and drug conjugates have been extensively studied [15,16], the use of co-administration of drugs with a cancer cell targeting ligand is less explored. Doxil, a liposomal formulation of chemotherapeutic DOX, is used clinically [20]. In addition, a peptide-drug conjugate (PDC) and several antibody-drug conjugates (ADC) are now approved for cancer treatment [16]. These drug conjugates target different cell-surface receptors that are overexpressed in cancer cells for specific receptor-mediated uptake of the conjugate. Coadministration of a targeting ligand with a drug also leads to increased uptake and efficacy. For instance, co-administration of peptide iRGD with various cytotoxic agents, such as doxorubicin, nab-paclitaxel (nanoparticles), or trastuzumab (antibody), was found to enhance the therapeutic efficacy of each of them [19]. This later strategy has advantages, such as no requirement for drug modification, which can reduce drug activity, and large amounts of drug can be delivered into the tumor tissue due to bystander effects [19]. 

**Figure 1:**
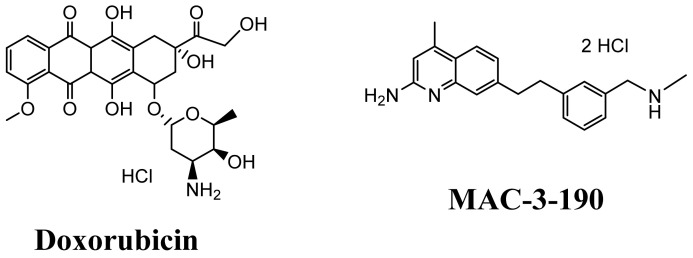
Structures of doxorubicin and MAC-3-190 used in this study.

We evaluated co-administration of a melanoma cell-specific peptide with DOX or MAC-3-190 (Figure 1) to improve their uptake by melanoma cells. In this study, we synthesized a peptide library on a functionalized cellulose membrane to screen for a peptide with specific affinity for melanoma cells, and low binding affinity to non-melanoma cells. We identified a 12-mer peptide, KK-11 (VPWXEPAYQRFL), for targeting melanoma; a D-amino acid substituted analogue of KK-11 (VPWxEPAYQrFL) was synthesized to improve its stability in serum. The peptide is specifically taken up by melanoma cells, both in vitro and in vivo. We then determined whether KK-11 improved the anti-melanoma activities of the tested drugs. Two types of anti-cancer drugs were studied, cytotoxic DOX and nNOS inhibitor MAC-3-190. The latter is a targeted therapy for melanoma [8,9]. Co-administration of KK-11 significantly enhanced the cytotoxicity of DOX in vitro, and the in vivo antitumor activity of MAC-3-190 in a melanoma xenograft mouse model [11]. Our study suggests that peptide KK-11 can be used as a carrier or targeting ligand in order to improve drug delivery to melanoma cells and enhance anti-melanoma activity.

## 2. Materials and Methods

### 2.1. Materials

Peptide array syntheses were conducted using an automated spot synthesis system (ResPep SL) as before (Intavis, Cologne, Germany) [21,22]. Individual peptide synthesis was performed on an automated peptide synthesizer (Tribute) from Protein Technologies (Tucson, AZ, USA). Purification of peptides used the Prominence-*i* HPLC System (Shimadzu, Kyoto, Japan). The ChemiDoc™ XRS+ system (Bio-Rad, Hercules, CA, USA) was used to record fluorescence intensity on the membranes.

Rink amide resin, L- and D- protected amino acid building blocks, and chemical reagents were purchased from AAPPTec. All solvents used for HPLC were obtained from Sigma-Aldrich, St. Louis, MO, USA, and used without further purification. VivoTag 680 XL (665/688 nm Excitation/Emission wavelength) was purchased from PerkinElmer. The targeted peptides were purified by RP-HPLC with a Shimadzu, C_18_ (19 × 250 mm) column, and the purity was confirmed by analytical RP-HPLC using a mobile phase composed of eluent A (99.9% *v/v* H_2_O and 0.1% *v/v* TFA) and eluent B (99.9% *v/v* CH_3_CN and 0.1% *v/v* TFA). Mass spectra were recorded with a Bruker Daltonics Autoflex MALDI-TOF using α-cyano-4-hydroxycinnamic acid (CHCA) as the matrix. ^1^H and ^13^C NMR spectra were obtained on a Bruker Avance III HDTM 400 NMR spectrometer (internal standard TMS), using deuterated methanol as solvent.

A Milli-Q system was used for ultrapure water. Sterile water was from Millipore Sigma. DMEM, Step/Pen, Hank’s Balanced Salt Solution (HBSS), 0.05% trypsin/EDTA, and horse serum were obtained from Gibco and Hyclone. All solvents and chemicals, including triisopropylsilane (TIPS), dichloromethane (DCM), dimethylformamide (DMF), *N*-methylmorpholine (NMM), diethyl ether, ethanol, acetonitrile, trifluoroacetic acid (TFA), diisopropylcarbodiimide (DIC), ethyl cyanohydroxyiminoacetate (Oxyma pure), *O*-(1*H*-6-chlorobenzotriazole-1-yl)-1,1,3,3-tetramethyluronium hexafluorophosphate (HCTU), and *N*,*N*-diisopropylethylamine (DIPEA) were obtained from Sigma-Aldrich. Methanol ACS was from EMD Millipore. Piperidine 20% was purchased from Protein Technology (Tucson, AZ, USA). CyQUANT and FITC dyes were obtained from Invitrogen (Eugene, OR, USA). Derivatized cellulose membranes were from Intavis (Cologne, Germany). Human serum was purchased from Sigma-Aldrich.

### 2.2. Cell Lines

Four human melanoma cell lines carrying distinct genomic mutations (A375: BRAF^V600E^/PTEN^WT^/CKIT^WT^; SK-Mel-28: BRAF^V600E^/PTEN^T167A^/CKIT^WT^; wm115: BRAF^V600E^/PTEN^WT^/CKIT^WT^; and WM3211: BRAF^WT^/PTEN^WT^/CKIT^L576P^) were used for the study [23,24]. A375, wm115, Sk-mel-28, and HEK-293 were purchased from American Type Culture Collection (ATCC; Manassas, VA, USA), and wm3211 was obtained from Rockland Immunochemicals (Limerick, PA, USA). Cell lines were cultured in Dulbecco’s Modified Eagle’s Medium (DMEM, #11995073; Gibco, Waltham, MA, USA) (A375) or Eagle’s Minimum Essential Medium (EMEM) (wm115, SK-Mel-28, HEK-293) with 10% fetal bovine serum (FBS, #26140079; Gibco, Waltham, MA, USA), or Tumor Specialized Media with 2% FBS (WM3211). Cell culture media were supplemented with 10% FBS (Corning, Corning, NY, USA). Cells were cultured in a humidified atmosphere in a 5% CO_2_ incubator maintained at 37 °C.

### 2.3. Peptide Array Synthesis

Fifty-three peptide sequences were synthesized in duplicate on derivatized cellulose membrane (Intavis, Cologne, Germany) using a ResPep SL Autospot robot. The detailed method was explained previously [21,22]. Briefly, the sequences of peptides were entered in the robot software, and 384 spot synthesis mode was selected for this library. First, the membrane was dipped in DMF for an hour followed by transfer and support to the holder of the robot on filter paper. Then, the membrane was washed with ethanol and subjected to a vacuum to remove any bubbles under the membrane. The Fmoc strategy was used for peptide synthesis. DIC and Oxyma pure were used as coupling reagent and racemization suppressor, respectively. After each coupling, capping of unreacted amino acids was done using acetic anhydride (3%), followed by 20% piperidine for Fmoc deprotection. The C-terminal ends of all peptides were bound to the membrane. Following the synthesis, the membrane was dipped in deprotection solution containing 15 mL TFA, 15 mL DCM, 900 µL TIPS, and 600 µL water, and kept for 3 h at room temperature. Next, membranes were thoroughly washed with DCM, DMF, and ethanol. Finally, they were air dried and kept in a sealed bag at −20 °C until use.

### 2.4. Peptide Array Cell Binding Assay

The sequential steps of the assay are as described in a previous publication [21,25]. The membrane was dipped in ethanol for 30 sec in order to remove any precipitation of hydrophobic peptides. Then, the membrane was dipped in sterile PBS at pH 7.4 for 30 min. The cells (75 × 10^3^ cells/mL) were seeded directly on the membrane in a sterile plate and incubated at 37 °C for 4 h. After washing off the unbound cells with sterile PBS, pH 7.4, the membrane was frozen at −80 °C for 2 h. Subsequently, it was defrosted at room temperature and incubated in CyQUANT solution at 37 °C for 30 min following the manufacturer’s protocol. The membrane was then washed with sterile PBS and air dried.

Membranes were scanned using the ChemiDoc™ XRS+ system (Bio-Rad, Hercules, CA, USA) to analyze the fluorescence intensity of each spot. The setting was adjusted based on the excitation and emission wavelengths of the CyQUANT dye at 465 nm and 535 nm, respectively. The fluorescence intensity (FI) was normalized to the FI of non-cancerous HEK-293 cells. The relative fluorescence densities were used for identifying peptides that exhibit high binding affinity to melanoma cells.

### 2.5. FITC Labeled-Peptide Synthesis and Purification

Four peptides, KK-1 KK-11, KK-12, and KK-13, were selected for uptake studies in human melanoma cells. These four peptides were synthesized based on Fmoc solid-phase peptide synthesis (Fmoc-SPPS) on preloaded Fmoc-Leu/Ala Wang resin (0.1 mmol scale) using an automated peptide synthesizer (Tribute, Gyros Protein Technologies, Uppsala, Sweden), and following the procedure reported previously [26,27]. Preloaded Fmoc leucine/alanine Wang resin (145/227 mg, 0.1 mmol) was added to the glass reaction vessel (RV). Resin swelling was done automatically with nitrogen blowing and mechanical shaking in DMF for 30 min. All amino acids were coupled in sequence using HCTU (2.5 equiv) and NMM (1.2 equiv) for each coupling and were mixed with amino acid (3 equiv) in 3 mL of DMF. Fmoc was removed with 20% piperidine in DMF automatically. β-Alanine was added at the N-terminus of the sequence as a spacer for FITC coupling. In the final step of the automated synthesis, an extra DCM washing was added to prepare resins for peptide cleavage. FITC (0.3 mmol) was mixed in 5 mL of DMF with DIPEA (Hunig’s reagent) (0.15 mmol) and then incubated with the resin in the dark for 20 h. The successful conjugation was confirmed using MALDI-TOF and RP-HPLC. Cleavage of peptides was done manually. The cleavage cocktail was 95% TFA (9.50 mL), 2.5% TIPS (250 µL), and 2.5% ultrapure water (250 µL). The peptides were precipitated using cold diethyl ether (20 mL). The purification was carried out using a semi-preparative RP-HPLC with a C18 Vydac column. The purity of the FITC-peptides was determined from analytical RP-HPLC chromatograms (AUC) and was found to be ≥95% (Figure S4). All peptides were characterized using MALDI-TOF mass spectrometry (Figure S5). The concentrations of peptides were obtained using the extinction coefficient of FITC. The absorption was read using a Shimadzu Nanodrop, and the concentration was calculated using the Beer-Lambert equation.

### 2.6. Synthesis of VivoTag-KK-11 Peptide

For the synthesis of fluorescent VivoTag 680 XL labeled D-amino acid analogue of KK-11 (VivoTag-KK-11), first, the linear peptide (D-amino acid analog of KK-11, D-aa KK-11) was synthesized following Fmoc SPPS on Rink amide resin (526 mg, 0.30 mmol, 0.57 mmol/g). After the synthesis, the crude targeted peptide was subjected to RP-HPLC for purification. The pure fraction was concentrated and subsequently freeze-dried to afford pure powdered peptide A**_β_**VPWxEPAYQrFL. The purity of the peptide was confirmed by analytical RP-HPLC, and the molecular weight by MALDI-TOF (*m*/*z*), [C_78_H_113_N_19_O_17_]: Calcd [M + H]^+^, 1588.9; Found [M + H]^+^, 1588.5 (Figure S2). Next, in an amber vial, D-aa KK-11 (2.00 mg, 0.0012 mmol) was dissolved in DMF (0.30 mL) followed by adding a solution of VivoTag 680 XL (0.57 mg, 0.0003 mmol, 0.3 equiv) in DMF (0.2 mL). The mixture was treated with Hunig’s reagent (3.12 μL, 0.018 mmol, 15 equiv) followed by stirring at room temperature for 2 h. After completion of the reaction, the solution was concentrated and added to cold ether, affording the green crude VivoTag-KK-11, which was subjected to analytical RP-HPLC for purification (R_t_ = 45 min). MALDI-TOF (*m/z*), [C_75_H_108_N_18_O_16_]: Calcd [M + H]^+^, 2823.2 Da; Found [M + H]^+^, 2823.4 Da (Figure S6).

### 2.7. Fluorescence Microscopy and Imaging

A375 melanoma cells were plated on coverslips and allowed to adhere overnight to 75% confluence, then incubated with 0.5 µM of FITC-KK-11 for 30 min at room temperature. After treatment, cells were washed three times in 1× PBS then fixed with 4% formaldehyde in 1× PBS for 15 min at room temperature. Fixed cells were then washed three times in 1× PBS for 5 min each, followed by curing in the dark with ProLong™ Gold Antifade Mountant with DAPI (P36935, Life Technologies Corporation, Eugene, OR, USA) for 1 h. Slides were visualized and recorded using the Nikon Eclipse Ti2-E confocal microscopy system (Nikon, Melville, NY, USA) using green and blue filters with 60× magnification. 

### 2.8. In Vitro Cellular Uptake Analysis

The peptide (FITC-labeled) concentration was determined using a Shimadzu BioSpec-nano Micro-volume UV-Vis spectrophotometer (Shimadzu, Kyoto, Japan). Human melanoma cells (wm3211, Sk-mel-28, and A375) were incubated with FITC or FITC-labeled peptides at final concentrations of 0.5 µM and 1 µM for 2 h. After thorough washing with cold 1× PBS, the melanoma cells in a single cell suspension were collected for flow cytometry analysis (BD FACSVerse, BD Biosciences, Franklin Lakes, NJ, USA). The mean fluorescence density of 10,000 cells was analyzed and compared to that of control cells, as described previously [22,27,28].

### 2.9. Serum Stability

Human serum (250 µL) was thawed to room temperature and was added to DMEM media (650 µL) [25]. The mixture was kept in a 37 °C incubator to mimic human body temperature for 15 min. The peptide (100 µL, 1 mM) was dissolved (dispersed) in sterile water. The peptide solution was added to the pre-warmed human serum and DMEM mixture. The mixture was incubated at 37 °C. At different time points (0, 0.5, 1, 5, and 24 h) aliquots (100 µL) were removed and added to methanol (200 µL). Each time point sample was kept at 4 °C for 5 min, and then was centrifuged at 500× *g* for 15 min to separate serum proteins. The supernatant was injected into the RP-HPLC, and the major peaks were collected. The peak’s mass was determined using MALDI-TOF mass analysis. The serum stabilities of the D-aa KK-11 were determined by comparing the HPLC peaks (AUC) for the intact peptides at different time points [25,29].

### 2.10. In Vitro Cytotoxicity Detected by MTT Colorimetric Assay

Cells were seeded in a 96-well plate and allowed to adhere overnight prior to adding the compound [29]. After 72 h of treatment in serum-free media, an MTT solution was added to each well to give a final concentration of 0.5 mg/mL and was incubated for 3 h. Formed crystals were solubilized, and their absorbance was measured at 595 nm.

### 2.11. Ex Vivo and In Vivo Imaging

The study was carried out in compliance with Animal Research: Reporting of In Vivo Experiments (ARRIVE) guidelines. All the animal procedures were approved by the Institutional Animal Care and Use Committee (IACUC) at Chapman University and conducted in compliance with the policies of Chapman University, in addition to those of federal, state, and local animal welfare authorities. Male and female athymic nude mice (4–6 weeks old) were kept (5 in each cage) in a pathogen-free environment. Approximately 1 million human melanoma A375 cells (0.1 mL in normal saline), mixed with 0.1 mL cold Matrigel (#354248; Corning, Corning, NY, USA), were injected subcutaneously into the flank. The tumor size was measured with a caliper, and body weight was recorded once a week. When the tumor size reached approximately 800–1000 mm^3^, as calculated using the following formula: [Length × (Width^2^)]/2 (in mm) [8], mice were processed for in vivo or ex vivo imaging [26].

For in vivo imaging, normal saline or VivoTag-KK-11 at a dose of 8 µg per mouse was injected into the mice via the tail vein (*n* = 2 for each time point). The animals were then scanned at different time intervals (0.5, 2, 6, and 24 h) using an IVIS Spectrum in vivo imaging system (PerkinElmer, Waltham, MA, USA). The mice were imaged for 0.5 s, 10 bin, level B at an emission wavelength of 688 ± 5 nm. Imaging was limited to no more than once a day and was conducted under continuously maintained isoflurane. Mice were maintained on alfalfa-free diets to minimize the background fluorescence. All the study animals survived through the in vivo imaging procedure.

For ex vivo imaging at defined time intervals (5, 15, 30 min, followed by 1, 1.5, 2, 3, 4, 6, 24, and 48 h) following fluorescence conjugate injection, animals were euthanized via carbon dioxide inhalation (*n* = 2 for each time point); tumor, liver, spleen, heart, lungs, brain, and kidneys were collected and briefly rinsed with saline. The organs were then imaged for 0.5 s, 10 bin, level B at an emission wavelength of 688 ± 5 nm using an IVIS Spectrum imaging system.

### 2.12. In Vivo Xenograft Melanoma Mouse Model 

The study was carried out in compliance with the ARRIVE guidelines. All the animal studies and procedures were approved by the IACUC at Chapman University and conducted in compliance with the policies of Chapman University, in addition to those of federal, state, and local animal welfare authorities. Nude mice (NU/NU) were purchased from Charles River (Wilmington, MA, USA) and were housed and maintained in the Chapman University vivarium under pathogen-free conditions. A375 cells were suspended in cold Matrigel and injected subcutaneously into the flank of each mouse (1 × 10^6^ cells per mouse) to establish tumors. The mice were treated by tail vein injections of 1× PBS, D-aa KK-11 alone (1.75 mg/kg), MAC-3-190 (5 mg/kg) alone, or D-aa KK-11 and MAC-3-190 co-treatment for 21 days. For the co-treatment, the solution of D-aa KK-11 and MAC-3-190 (1:11 molar ratio) was mixed gently and incubated at room temperature for 30 min before tail vein injection. The growth of the tumors was monitored three times a week and measured using digital Vernier calipers. The tumor volume (mm^3^) was calculated as [Length × (Width^2^)]/2 (in mm) [8]. The mice were sacrificed after 21 days per IACUC policy when the control group’s average tumor size reached 2000 mm^3^. Tumor xenografts were removed and weighed.

### 2.13. Statistical Analyses

All in vitro experiments were repeated at least twice and performed in at least two different human melanoma cell lines. Data shown are means ± SD from a representative of at least two independent experiments. Statistical analysis was performed using the Student’s *t*-test, and a *p*-value of less than 0.05 was considered statistically significant. 

## 3. Results

### 3.1. Peptide Library Synthesis and Screening

**Figure 2:**
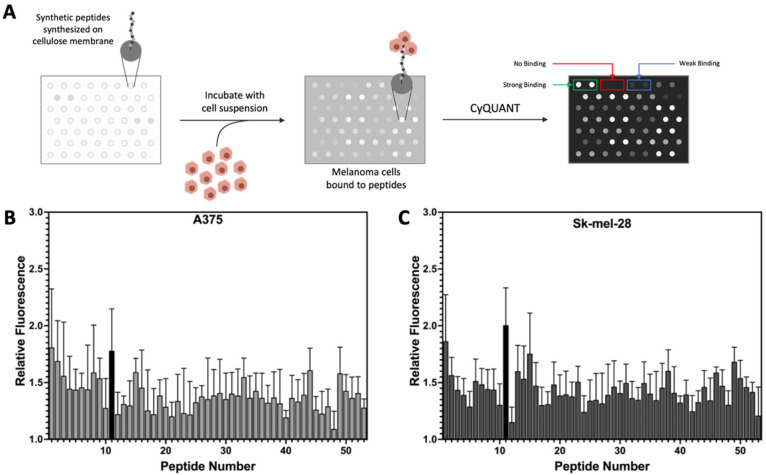
Peptides for targeted delivery in human melanoma. (**A**) Schematic showing peptide array screening for melanoma specific peptides. The average CyQUANT fluorescence intensity of the peptide bound cells was used to estimate the relative cells bound to the peptides. (**B**,**C**) Relative fluorescence was determined compared to human embryonic kidney cells (HEK-293). Each bar represents one peptide synthesized in the library as listed in Table 1. The top-ranked peptides (such as **11**, black) showing a better binding profile for melanoma cells (**B**, A375 cells; **C**, Sk-Mel-28 cells) were further labeled with FITC for cellular uptake analysis.

A library of 53 peptides was synthesized in an array format on a cellulose membrane using SPOT synthesis (Table 1) [21]. The peptide library was designed starting with five lead cancer cell-targeting peptides, Arg peptide (**1**, Table 1), p160 analogue (**11**), RGD (**16**), GE11 (**20**), and LSD (**37**) [21,30,31,32,33]. Conservative substitutions, deletion from the N- or C-terminus, alanine scan, and scrambling of amino acids were used to design the analogues for the library. The peptides were screened for specific binding to three human melanoma cell lines. The cells were incubated with the cellulose membranes with conjugated peptides followed by incubation with CyQUANT fluorescence dye. A brief schematic for the peptide array-whole cell binding assay for screening peptides with high affinity for melanoma cells is shown in Figure 2A. The relative cell adhesion capacities of the peptides to A375 (Figure 2B), Sk-mel-28 (Figure 2C), and wm115 (Figure S3b, Supporting Information) were estimated based on the fluorescence of the bound melanoma cells compared to normal human HEK-293 cells (Figure S3a). Two peptides, **1** and **11**, showed high specificity for melanoma cells, as evidenced by the highest fluorescence ratio for melanoma to non-melanoma cell binding (Table 1).

### 3.2. Synthesis of Soluble Fluorescently Labeled Peptides

Soluble peptides labeled with fluorescent FITC were synthesized to evaluate uptake in melanoma cells. FITC was attached to the N-terminus of the peptides, and β-alanine was used as a spacer between the peptide and FITC. Based on the binding studies, four peptides, called KK-1, KK-11, KK-12, and KK-13, were synthesized (Table 1). Peptides **12** and **13** were used as control sequences. The peptides were synthesized using solid phase methodology according to the previously reported procedure [25]. FITC-labeled peptides were purified using RP-HPLC (purity ≥ 95%, Figure S4A–D), and were characterized using MALDI-TOF mass spectrometry (Figure S5A–D, Table 2). Pure peptides were dried and stored at −20 °C until use.

**Scheme 1:**
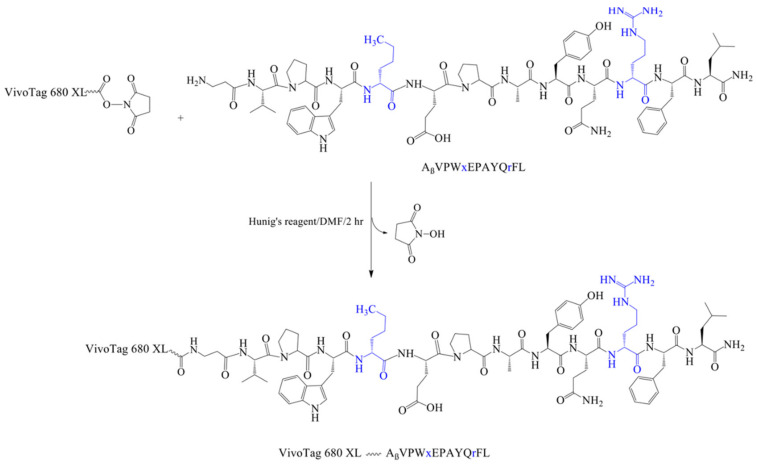
Synthesis of VivoTag-KK-11. Steps for conjugating VivoTag 680 XL to D-aa KK-11 using N-terminal β-alanine as a spacer following the manufacturer’s protocol. Blue color and lower-case letters stand for D-amino acids, and X stands for norleucine.

For in vivo studies, peptide KK-11 was labeled with a fluorescent Vivotag 680 XL in the N-terminus (Scheme 1, Table 2). In addition, two amino acids in the sequence (norleucine and arginine) were substituted with D-amino acids in order to increase proteolytic stability (Table 2). These amino acids were identified as proteolytically labile sites in our previous study [25]. The D-aa KK-11 peptide was assembled on acid–labile Rink amide resin (Supporting Information) and was characterized (Figure S1). The proteolytic stability of the D-aa KK-11 peptide was evaluated by incubating with human serum. The presence of the intact peptide after incubation with human serum for different time periods was detected using RP-HPLC and confirmed by MALDI-TOF mass analysis. Our data showed that D-aa analog of KK-11 exhibited greatly improved stability in serum for up to 5 h (Figure S7). The area under the curve of the peptide peak at 5 h was 97.4% of control (t = 0 h), which was reduced to 45.3% by 24 h. However, peptide KK-11 with all L-amino acids reduced to an undetectable level within 30 min (data not shown). For conjugating VivoTag 680 XL dye, the peptide was modified by incorporating a β-alanine moiety, which generated the precursor A_β_-KK-11. The structure and mass of A_β_-KK-11 were confirmed by NMR (^1^H and ^13^C) and MALDI-TOF [M + H]^+^ 1588.5 Da (Figure S2). Next, the β-alanine terminal amino group was allowed to react with the succinimidyl ester group of the reactive fluorophore (VivoTag 680 XL) under basic conditions in polar aprotic solvent, generating the fluorescently labeled peptide with extrusion of N-hydroxysuccinimide (NHS) (Scheme 1). The mass of the fluorescence-labeled VivoTag-A_β_-KK-11 was confirmed by MALDI-TOF, showing a peak at 2823.4 Da, corresponding to [M + H]^+^ (Figure S6, Table 2).

### 3.3. Uptake of Select Peptides by Melanoma Cells

**Figure 3:**
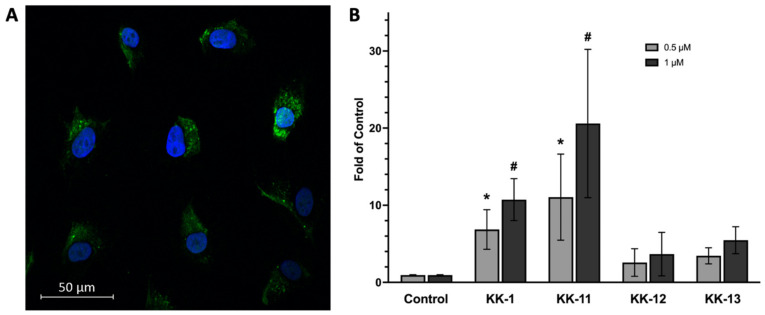

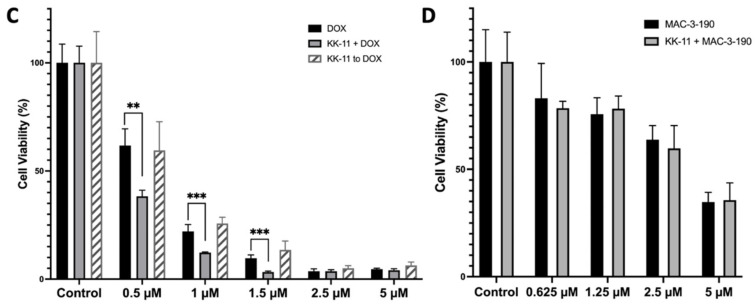
Uptake and drug delivery of KK-11 in human melanoma cells. (**A**) A representative fluorescence microscopy image of A375 melanoma cells after incubation with FITC-KK-11 (0.5 µM) for 30 min at 37 °C. Cell nuclei were stained blue with DAPI. (**B**) Uptake of FITC-peptides KK-1, KK-11, KK-12, and KK-13 by human melanoma cells. The bar represents the average fluorescence density of three cell lines (A375, Sk-mel-28, and wm3211 cells) detected by fluorescence-activated cell sorting (FACS) analysis. The cells were incubated with FITC-labeled peptide for 30 min at 37 °C (0.5 µM, grey bar; 1 µM, dark bar). The control is cells were incubated with FITC alone. The bar graph shows mean fluorescence intensity (MFI) of three experiments ± SD; *, *p* < 0.05 compared to 0.5 µM FITC alone; #, *p* < 0.001 compared to 1 µM FITC alone. (**C**) Co-treatment of KK-11 peptide enhanced the cytotoxicity of DOX in melanoma A375 cells. Cells were either treated with DOX or co-treated with DOX and KK-11 (1 µM) for 72 h (KK-11 + DOX), or pretreated with KK-11 for 4 h, then washed away following a DOX 72-hour treatment (KK-11 to DOX). Control is DMSO or KK-11 alone. Cell viability was detected by an MTT colorimetric assay. Statistical analysis was performed with a Student’s *t*-test, and error bars denote mean ± SD; **, *p* < 0.01; ***, *p* < 0.001 compared to DOX alone. (**D**) Co-treatment with KK-11 did not enhance the cytotoxicity of nNOS inhibitor MAC-3-190 in A375 melanoma cells. Cells were treated with MAC-3-190 at various concentrations with or without 1 µM KK-11 for 72 h. Cell viability was detected by MTT assay. The assays were repeated independently at least three times.

As shown in the fluorescence microscopy imaging (Figure 3A), FITC-KK-11 (Table 2) was present in the cytoplasm of A375 melanoma cells after 30 min incubation at 0.5 µM concentration. The uptakes of FITC-labeled KK-11 and three other peptides (KK-1, KK-12, and KK-13) were further confirmed using flow cytometry analysis (Figure 3B). Among the four tested peptides, the uptake of KK-11 by melanoma cells was observed to be the highest, and in a dose-dependent manner. At 1 µM concentration, the average fluorescence intensity was increased to more than 20-fold of FITC alone (control). KK-1 peptide also showed significantly increased uptake in melanoma cells, but to a lesser extent compared to KK-11 at the same concentration.

### 3.4. KK-11 Co-Treatment Significantly Enhanced the Cytotoxicity of DOX in Human Melanoma Cells

We further determined whether KK-11 enhances the drug delivery of cytotoxic DOX and nNOS inhibitor MAC-3-190 in vitro. Human melanoma A375 cells were then incubated with DOX in the presence or absence of KK-11. As shown in Figure 3C, DOX (0.5 µM) alone reduced cell viability to 62% of control. Co-treatment with KK-11 (1 µM) significantly enhanced the cytotoxicity of DOX and decreased cell viability to 40% of control (*p* < 0.001). At 1 µM, DOX in combination with KK-11 reduced cell viability to 14% of control cells, while 24% of melanoma cells survived from DOX treatment alone. Of note, treatment of melanoma cells with KK-11 and DOX sequentially failed to produce any changes on DOX cytotoxicity in melanoma cells.

The effects of KK-11 co-treatment (1 µM) with nNOS inhibitor MAC-3-190 were studied at different concentrations (25% IC_50_, 50% IC_50_, IC_50_, and 2× IC_50_). However, we did not observe any significant enhancement of cytotoxicity in the presence of KK-11 compared to MAC-3-190 treatment alone (Figure 3D).

### 3.5. Bio-Distribution of KK-11 in Tumor Xenografts

**Figure 4:**
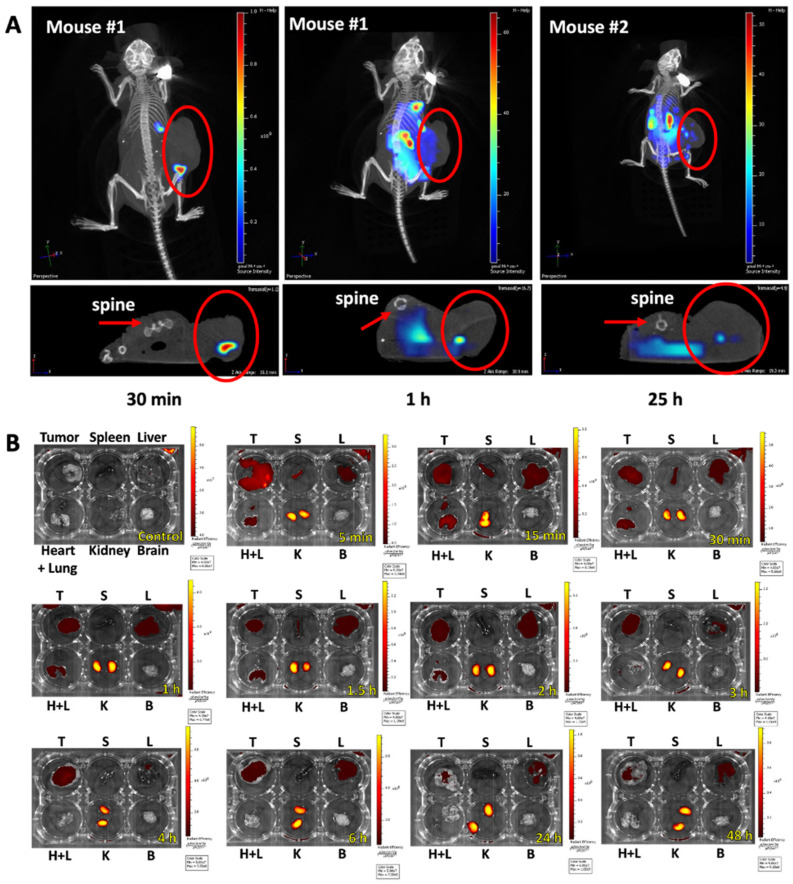
In vivo and ex vivo organ distribution images of VivoTag 680 XL-labeled D-aa KK-11 in athymic nude mice bearing human melanoma A375 tumors. Saline or VivoTag-KK-11 in a dose of 8 µg per mouse was injected into the mice via the tail vein (*n* = 2 for each time point). (**A**) Representative images of CT-fluorescence scans at tumor area sections were captured in live mice at different times after injection (30 min, 1 h, or 25 h). The top panels represent the scanned images of the tumor section area in coronal views. The bottom-row pictures display the fluorescence image in transaxial views: arrows point to the spine, and circles mark the xenograft tumors. (**B**) At defined time intervals (5 min, 15 min, 30 min, 1 h, 1.5 h, 2 h, 3 h, 4 h, 6 h, 24 h, and 48 h) following peptide injection, animals were euthanized. Organs, including tumor xenografts (T), liver (L), spleen (S), heart and lungs (H + L), kidneys (K), and brain (B), were excised and imaged using the IVIS Spectrum imaging system. Representative fluorescence images of different organs show peptide distribution at different time points as indicated.

Armed with this specific melanoma-binding peptide, we further studied the effects of KK-11 on drug delivery and antitumor efficacy. First, D-aa KK-11 was conjugated to VivoTag 680 XL fluorescence tag (Scheme 1) to visualize the in vivo distribution in athymic mice bearing human melanoma xenografts. After injection via tail vein, the mice were then taken for in vivo imaging using the IVIS CT-Fluorescence imaging system. As shown in Figure 4A, 30 min after injection, VivoTag-KK-11 distributed to the xenograft tumor and accumulated in the spotted area. The fluorescence remained detectable in tumor xenografts 25 h after the administration of VivoTag-KK-11.

Further ex vivo studies demonstrated that the peptide distributed to the tumor and to several other organs nonspecifically within 5 min, including the lungs, heart, and spleen (Figure 4B), but cleared readily from other organs by 6 h after injection. The ex vivo imaging of excised tumor xenografts confirmed the presence of the peptide in tumor tissue. The fluorescence of VivoTag-KK-11 was evident as early as 5 min after IV injection and remained detectable up to 24 h post injection in tumor xenografts. This finding was consistent with the in vivo imaging study in live mice (Figure 4A). After 6 h, VivoTag-KK-11 was predominantly detected in kidneys up to 48 h after injection, where the peptide is metabolized. Of note, no brain penetration was observed. The preferential and prolonged accumulation of D-aa KK-11 in the tumor suggests that it may enhance the targeted delivery of anticancer drugs to melanoma tumors, which will ultimately improve their antitumor activities.

### 3.6. Co-Administration of a Melanoma-Targeted Peptide D-aa KK-11 Enhanced the Antitumor Activity of nNOS Inhibitor MAC-3-190

**Figure 5:**
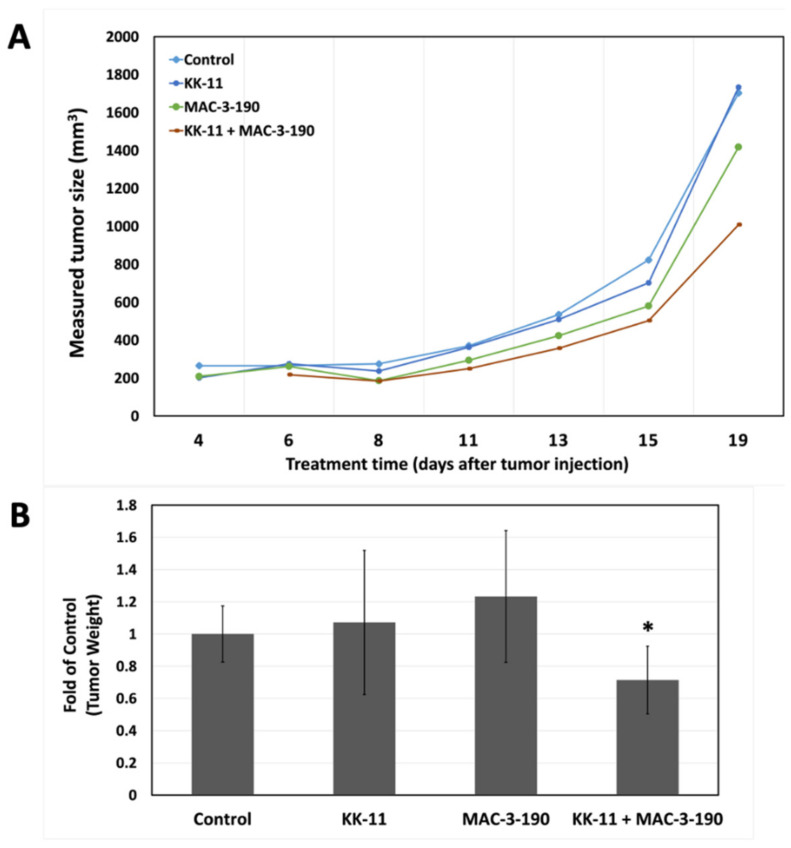

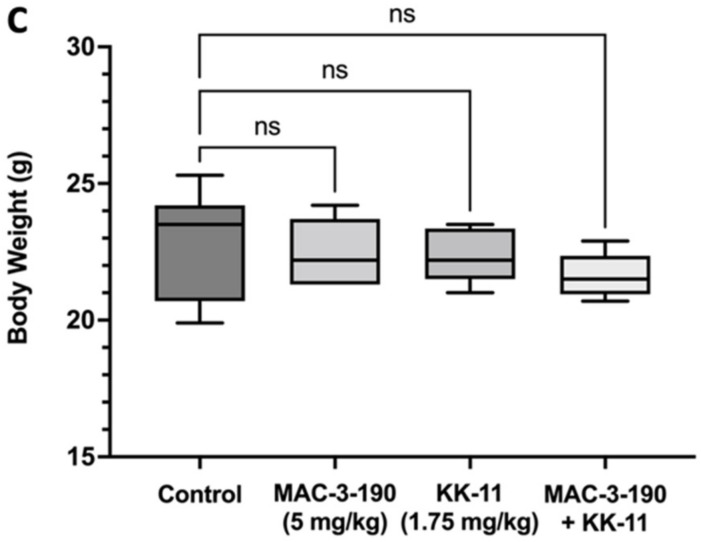
Enhanced antitumor activity of nNOS inhibitor MAC-3-190 co-injected with D-aa KK-11 peptide in a xenograft mouse model. Mice bearing human melanoma A375 tumors were intravenously injected with either D-aa KK-11, MAC-3-90, or a mixture of MAC-3-190 (5 mg/kg) and D-aa KK-11 (1.75 mg/kg) or 1× PBS, once daily. (**A**) The tumors were measured three times a week using digital Vernier calipers. Tumor volume (mm^3^) was calculated as [Length × (Width^2^)]/2. (**B**) The tumors were harvested and weighed after 21 days of treatment (Control: *n* = 10; D-aa KK-11 alone: *n* = 5; MAC-3-190: *n* = 5; D-aa KK-11 + MAC-3-190: *n* = 10). (**C**) The body weights of the mice were measured after 21 days of treatment. Statistical analysis was performed with Student’s *t*-test, and error bars denote mean ± SD; *, *p* < 0.05 compared to control group.

In vitro, we did not observe any significant difference in cytotoxicity between MAC-3-190 and co-administration of KK-11 and MAC-3-190 (Figure 3D). In addition, our previous studies and preliminary observations demonstrated that MAC-3-190 (5 mg/kg/day) does not have any significant antitumor activity in vivo [8]. Therefore, we evaluated if co-administration with KK-11 peptide would affect the antitumor activity of MAC-3-190 in vivo in mice carrying melanoma xenografts. Using an A375 melanoma xenograft mouse model, we compared the antitumor activity between mice injected with MAC-3-190 (5 mg/kg) and mice injected with a mixture of D-aa KK-11/MAC-3-190. Mice were also injected with saline or D-aa KK-11 to serve as controls. As shown in Figure 5A, co-administration of D-aa KK-11 with MAC-3-190 (1:11 molar ratio) exhibited significant inhibitory effects after 21-day treatment on tumor growth compared to the MAC-3-190 (5 mg/kg) and control groups. Co-treatment reduced tumor growth by 28.6% (*p* < 0.05; Figure 5B) without remarkable systemic toxicity occurring as indicated by the body weight at the end of 21 days of treatment (Figure 5C). MAC-3-190 was used in excess, as peptide can deliver higher concentrations of the drug to the cancer site with a higher drug-to-peptide ratio (DPR). For similar reasons, a peptide-drug conjugate, ANG1005 with a PDR of 3, is currently in clinical trials for paclitaxel delivery [34]. Also, the approved ADCs typically have a drug-to-antibody ratio (DAR) of 3–8 [16].

## 4. Discussion

Cutaneous melanoma is the most aggressive and difficult to treat skin cancer. Traditional chemotherapeutics are nonspecific, and produce a very limited response in melanoma patients often accompanied by toxicity and severe myelosuppression [35]. Novel approaches to improve available melanoma therapeutics, including organic and inorganic nanomaterials, have been developed for drug delivery, such as liposomes, polymers, dendrimers, and micelles [36]. Different nanomaterials offer various advantages, including controlled release, reduced systemic toxicity, and protection from metabolic inactivation. The use of nanoparticles, however, has been limited due to concerns regarding in vivo distribution, immunogenicity, limited tissue penetration and stability, rapid removal, and degradation [37].

In recent years, more efforts have focused on conjugation to cancer cell-targeting ligands, such as monoclonal antibodies (mAbs) and peptides, to improve targeted delivery of anti-cancer drugs or nanoparticles [16,38,39,40,41,42,43]. mAbs are attractive delivery vehicles as a result of their high target specificity and affinity. However, mAbs possess significant limitations. Due to their complexity and size, mAbs have limited tissue penetration, high production costs, and a high risk of immunogenicity [44,45,46]. In addition to target expression and internalization, drug loading and conjugation are essential factors to consider in antibody-drug conjugates (ADCs) [47]. Different ratios have been found to affect the pharmacokinetic properties, therapeutic index, and antigen binding [48].

Peptide-drug conjugates (PDCs) are an appealing alternative to ADCs and may offer solutions to the limitations posed by mAbs [16,49]. Short peptides have low oral bioavailability but are easily synthesized to homogeneity and allow for considerable flexibility resulting from the diversity of amino acid combinations, which enable the alteration of physiochemical properties, specificity, and stability, generally without immunogenic responses [29,50,51]. PDCs have shown promising anti-cancer activities in many difficult-to-treat malignancies, such as pancreatic cancer [52] and melanoma [17]. The Kaur group is developing peptide-doxorubicin conjugates in order to treat triple-negative breast cancer (TNBC) by targeting cell-surface keratin-1 (K1) or epidermal growth factor receptor (EGFR) in cancer cells [22,28,29,53]. The success of PDCs is also evident from the FDA approval (2018) of a peptide conjugate, lutetium 177 DOTA-TATE, where radionuclide ^177^Lu is covalently linked to a somatostatin receptor-targeting peptide [54]. This PDC is approved for treatment of somatostatin receptor-positive gastroenteropancreatic neuroendocrine tumors.

In our study, peptide KK-11 was identified to specifically bind to wm115 (primary), A375 (metastatic), and Sk-mel-28 (metastatic) melanoma cell lines compared to normal HEK-293 cells (Figure 2 and Figure S3). FITC-labeled KK-11 also demonstrated dose-dependent uptake in melanoma cells, suggesting KK-11 can be a sufficient carrier to improve the drug delivery to melanoma (Figure 3). However, further studies are warranted to determine the selectivity of KK-11 in melanoma over non-melanoma cells. In our preliminary study, we observed that KK-11 can also bind to human primary glioma U87 cells (data not shown here). Further, structural optimization via D-amino acid substitution significantly improved serum stability (Figure S7). After a single injection, the peptide (VivoTag -KK-11) remains detectable in the tumor xenografts for at least 24 h, as shown in our in vivo and ex vivo biodistribution analyses (Figure 4). Co-treatment with KK-11 was shown to enhance the cytotoxicity of DOX significantly (Figure 3C). However, such effects of KK-11 were not evident after sequential treatment. These results indicate that KK-11 may serve as a targeted delivery vehicle and form a noncovalent complex with DOX as the cargo, shuttling it into the cell to enhance DOX cytotoxicity in A375 melanoma cells. In future, we plan to determine if the D-aa KK-11 complex could improve the anti-melanoma efficacy of DOX in vivo. Previous studies also showed that co-administration of a tumor targeting peptide iRGD with chemotherapeutic agents, such as doxorubicin, nab-paclitaxel (nanoparticles), or trastuzumab (antibody), enhanced the efficacy of cancer drugs [19].

The use of peptides as delivery vehicles can be achieved through covalent conjugation or physical complexation of the targeting peptide to its cargo [19]. Covalent conjugation requires expertise in peptide-receptor interactions in order to optimize the site of drug attachment without affecting drug potency and receptor binding, in addition to the selectivity of the peptide via steric hindrance [55]. A proper bond or linker should not affect the affinity and specificity of the peptide, and should render the drug conjugate stable until it reaches the target where the drug can be released [56]. Physical complexation is readily executed by simply mixing the targeting peptide with the cargo drug; however, the formation of noncovalent interactions is dependent on the physicochemical properties of the two interacting components and the formulation [57]. This approach tolerates varying molar mixing ratios; however, it also produces a mixture of nonhomogeneous structures [58]. In our future studies, VivoTag-labeled peptides will be co-administered with Cy.7-labeled MAC-3-190, followed by murine in vivo imaging, to track the biodistribution of both the peptide and the drug at different time intervals. The drug levels reached in the tumor will also be evaluated in the presence or absence of targeting peptides.

Small molecule drugs possess advantages, including favorable pharmacokinetics, low production costs, and high patient compliance [59]. Our previous studies have shown that neuronal nitric oxide synthase (nNOS) expression levels in patient biopsies significantly correlated with the disease stage, and that nNOS inhibitors may be a promising direction for melanoma treatment [9,10]. We found that several small molecule nNOS inhibitors, such as MAC-3-190 (Figure 1), exhibit potent and promising anti-melanoma activities [8,9,11]. However, nitric oxide (NO) is involved in regulating muscle tone in the sphincter of the lower esophagus, pylorus, sphincter of Oddi, and anus [60]. Gastrointestinal changes were reported in animal studies after nNOS inhibitor treatment, with delayed gastric emptying and colonic transit [61]. In a recent study, antibiotics were found to alter the expression of nNOS in the murine gut, resulting in similar observations along with an increase in the thickness of muscularis externa in the stomach, ileum, and cecum [62]. Enlarged stomachs with hypertrophy of the pyloric sphincter were also observed in transgenic mice with homozygous depletion of the nNOS gene [63]. In our current animal study (Figure 5C), the median body weight in the MAC-3-190 treatment group was lower compared to the control group but was not statistically significant (*p* > 0.05). Given the potential gastrointestinal adverse effects associated with nNOS inhibition, our strategy aims to blend targeted therapy (nNOS inhibitors) with targeted delivery, by coupling the small molecule with KK-11 to optimize the anti-melanoma treatment. We hope that by using this approach, we may further lower the effective dosage of nNOS inhibitors without compromising their anti-tumor activities. In this study, we examined whether KK-11 could enhance the in vivo anti-melanoma activity of a small molecule nNOS inhibitor MAC-3-190 (Figure 5). Currently, pharmacokinetic studies to determine the drug levels in tumor tissues and normal organs are underway.

In contrast to the effects observed in A375 cells with doxorubicin treatment (Figure 3C), co-incubation of KK-11 with MAC-3-190 did not enhance cytotoxicity in vitro (Figure 3D). However, our in vivo study demonstrated that co-administration of KK-11 with the selective nNOS inhibitor MAC-3-190 significantly reduced tumor growth compared to control and MAC-3-190 alone (Figure 5A), while this antitumor activity was not observed with low-dose MAC-3-190 treatment alone. Of note, the co-treatment group’s body weight was lower compared to that of the groups treated with MAC-3-190 alone or the vehicle control (Figure 5C). However, such a difference was not statistically significant by ordinary one-way ANOVA analysis. No cytotoxicity of KK-11 alone was observed in our in vitro or in vivo studies.

## 5. Conclusions

Our study suggests that in mixtures with drugs, peptide KK-11 enhances the cytotoxicity of anti-cancer drugs by acting as a specific melanoma-targeting drug carrier both in vitro and in vivo. When used in vivo, D-aa KK-11 likely accumulates the anti-cancer drug MAC-3-190 in the tumor microenvironment and within the melanoma cells, as evidenced by significant tumor volume reduction in mice treated with D-aa KK-11/MAC-3-190 compared to mice treated with MAC-3-190 alone. Further investigation is warranted to determine the optimal dose and ratio for a D-aa KK-11 and MAC-3-190 combination treatment. Further, covalent peptide-drug conjugates of D-aa KK-11 and MAC-3-190 with different linkers can be synthesized for bioactivity screening and study.

The binding target of KK-11 in melanoma cells is not yet defined. A pull-down assay to capture the protein that specifically interacts with KK-11, followed by protein sequencing, is warranted. Determining the protein to which KK-11 binds will allow for further structure-based optimization of KK-11 to improve its binding and uptake into melanoma cells and, ultimately, optimize the anti-melanoma therapy. The uptake mechanism is likely receptor-mediated endocytosis, based on our previous studies with breast cancer (MCF-7 and MDA-MB-435) cells [21,27,53]. However, this needs to be verified for melanoma cells. Our multifaceted approach used targeted delivery (via a peptide ligand) and targeted therapy (nNOS inhibitors), which will help lessen side effects and reduce the dose of the antitumor medication by facilitating drug delivery and accumulation within tumors.

## Figures and Tables

**Table 1:** Peptide library screening for melanoma cell binding. Based on our previous studies and published literature, a library containing 53 peptides in duplicates was designed and synthesized on cellulose membranes using an automated spot synthesis ResPep SL apparatus (Intavis, Cologne, Germany). The lead peptides for the library design were Arg peptide (**1**), p160 analogue (**11**), RGD (**16**), GE11 (**20**), and LSD (**37**). X stands for norleucine.

Peptide Number	Amino Acid Sequence	Fluorescence Ratio (Melanoma/Non-Melanoma Cells) *	Peptide Number	Amino Acid Sequence	Fluorescence Ratio (Melanoma/Non-Melanoma Cells) *
1	GRRPRPRPRP	1.82	28	YHWYGY**A**PQNVI	1.26
2	GRRPRPRPRPW	1.60	29	YHWYGYT**A**QNVI	1.32
3	GRRPRPRPRPWW	1.50	30	YHWYGYTP**A**NVI	1.30
4	GRRPRPRPRPWWW	1.46	31	YHWYGYTPQ**A**VI	1.33
5	GRRPRPRPRPWWWW	1.43	32	YHWYGYTPQN**A**I	1.29
6	G**A**RPRPRPRP	1.37	33	YHWYGYTPQNV**A**	1.35
7	GR**A**PRPRPRP	1.47	34	YHWYGYTP**E**NVI	1.36
8	GRRP**A**PRPRP	1.57	35	YHWYGYTPQ**D**VI	1.35
9	GRRPRP**A**PRP	1.51	36	YHWYGYTPQ**K**VI	1.23
10	GRRPRPRP**A**P	1.31	37	CLSDGKRKC	1.29
11	VPWXEPA**YQRF**L	1.80	38	**A**GRKLDSK**A**	1.39
12	WXEA**AYQRF**L	1.31	39	**A**DRSKGKL**A**	1.27
13	EPA**AYQRF**TA	1.39	40	**A**LSDGKRK**A**	1.23
14	RVPWLEAPYA	1.42	41	**A**LSDGKRKC	1.32
15	FVPWLEAPYA	1.61	42	C**A**SDGKRKC	1.25
16	RGDAAYQRFL	1.47	43	CL**A**DGKRKC	1.26
17	RGEPAYQRFL	1.30	44	CLS**A**GKRKC	1.44
18	RGEPAYQGRFL	1.24	45	CLSD**A**KRKC	1.22
19	RGDPAYQGRFL	1.35	46	CLSDG**A**RKC	1.35
20	YHWYGYTPQNVI	1.20	47	CLSDGK**A**KC	1.27
21	WQTNYIHPYVYG	1.21	48	CLSDGKR**A**C	1.12
22	YGPWYNHYITQV	1.28	49	CLSDGKRK**A**	1.57
23	**A**HWYGYTPQNVI	1.31	50	CLS**E**GKRKC	1.38
24	Y**A**WYGYTPQNVI	1.15	51	CLSDG**R**RKC	1.32
25	YH**A**YGYTPQNVI	1.28	52	CLSDGKR**R**C	1.27
26	YHW**A**GYTPQNVI	1.29	53	**W**LSDGKRKC	1.12
27	YHWYG**A**TPQNVI	1.19			

* Fluorescence ratio is the average ratio of fluorescent intensity of melanoma cancer cells (A375, Sk-mel-28, and wm115) bound to a peptide divided by that of non-melanoma HEK-293 cells bound to the same peptide.

**Table 2:** Sequence and MALDI-TOF mass analysis of different KK-11 peptides used in the study.

Peptide	Label	[M + H]^+^	
		Calculated	Found
VPWXEPAYQRFL	KK-11	1517.8	1518.1
VPWxEPAYQrFL	D-aa KK-11	1517.8	1517.6
FITC-A_β_-VPWXEPAYQRFL	FITC-KK-11	1979.2	1979.1
VivoTag-A_β_-VPWxEPAYQrFL	VivoTag-KK-11	2823.2	2823.4

## Supplementary Materials

The following supporting information can be downloaded at: https://www.mdpi.com/article/10.3390/pharmaceutics14051036/s1, Figure S1: Peptide D-aa KK-11 (a) chemical structure, (b) H^1^ NMR, (c) C^13^ NMR, and (d) mass spectrum, and (e) analytical RP-HPLC chromatogram; Figure S2: Peptide D-aa A_ß_-KK-11 (a) chemical structure, (b) H^1^ NMR, (c) C^13^ NMR, (d) mass spectrum, and (e) analytical RP-HPLC chromatogram; Figure S3: (a) Screening of peptide library (53 peptides in duplicates) for binding to HEK-293 cells; (b) Selective binding of peptides to human primary melanoma wm115 cells compared to HEK-293 cells. The binding fluorescence ratio of wm115 to HEK-293 represents the relative selectivity of peptide bindings with melanoma cells; Figure S4: Analytical RP-HPLC chromatogram of FITC labelled peptides, (a) KK-1, (b) KK-11, (c) KK-12, and (d) KK-13. Gradient used on a Vydac C18 analytical column was as shown with 0.1%TFA in water as Sol A and 0.1% TFA in acetonitrile as solvent B, and the absorbance was monitored at 220 nm; Figure S5: MALDI-TOF mass spectra for FITC labelled peptides; Figure S6: Peptide VivoTag 680 XL-A_ß_-KK-11 (a) chemical structure, (b) mass spectrum, and (c) analytical RP-HPLC chromatogram; Figure S7: D-amino acid analogue of KK-11 exhibits increased serum stability. To prepare, 100 µL of 1 mM of tested peptide was dissolved in sterile water and was added to pre-warmed DMEM medium containing 25% human serum. After vortexing, 100 µL of the final solution was added to 200 µL of methanol for RP-HPLC analysis after different incubation periods.
