# Supplementary material for: A Small Peptide Increases Drug Delivery in Human Melanoma Cells
Source: Pharmaceutics. 2022 May 11;14(5):1036. doi: 10.3390/pharmaceutics14051036 (PMC9145755; doi:10.3390/pharmaceutics14051036)
Supplement: Supplementary file 1 [file pharmaceutics-14-01036-s001.zip › pharmaceutics-1695896-supplementary.pdf]

Supporting Information

A Small Peptide Increases Drug Delivery in Human Melanoma Cells

Shirley Tong,[1] Shaban Darwish,[2,#] Hanieh Hossein Nejad Ariani,[2] Kate Alison Lozada,[1] David Salehi,[2] Maris A. Cinelli,[3,§] Richard B. Silverman,[3,4] Kamaljit Kaur,[*, 2] and Sun Yang[*, 1]

[1] *Department of Pharmacy Practice, Chapman University School of Pharmacy, Irvine, California, USA;*

[2] *Department of Biomedical and Pharmaceutical Sciences, Chapman University School of Pharmacy, Irvine, California, USA;*

[3] *Department of Chemistry, Department of Molecular Biosciences, Chemistry of Life Processes Institute, Center for Developmental Therapeutics, Northwestern University, Evanston, IL, USA;*

[4] *Department of Pharmacology, Feinberg School of Medicine, Northwestern University, Chicago, IL, USA*

[#] *Current address: Organometallic and Organometalloid Chemistry Department, National Research Centre, El Bohouth st, Dokki, Giza, Egypt*

[§] *Current address: Department of Medicinal Chemistry and Biological Chemistry, Northern Michigan University, Marquette, MI, USA*

Corresponding Author

Sun Yang, Bs.Pharm, Ph.D., BCPPS, APh

Chapman University School of Pharmacy

Harry and Diane Rinker Health Science Campus

9401 Jeronimo Road, Irvine, CA 92618, USA

Tel: (714) 516-5418

E-mail: syang@chapman.edu

Co-corresponding Author

Kamaljit Kaur, Ph.D.

Chapman University School of Pharmacy

Harry and Diane Rinker Health Science Campus

9401 Jeronimo Road, Irvine, CA 92618, USA

Tel: (714) 516-5494

E-mail: kkaur@chapman.edu

1. Fmoc-VPWxEPAYQrFL (D-aa Fmoc-KK-11)

Fmoc was deprotected from the Rink amide resin using piperidine in DMF (20% *v/v*). The solvent was drained, followed by coupling Fmoc-Leu-OH (318 mg, 0.9 mmol, 3 equiv) using HBTU (341 mg, 0.9 mmol, 3 equiv) and DIPEA (312 µl, 1.8 mmol, 6 equiv) in anhydrous DMF (10 mL) for 1 h. The resin was washed three times using DMF (10 mL x 3), followed by deprotection of Fmoc group by previously prepared piperidine solution. The resin was washed with DMF three times (10 mL x 3) to be ready for the next coupling. The subsequent amino acids, Fmoc-Phe-OH (348 mg, 0.9 mmol, 3 equiv), Fmoc-D-Arg(Pbf)-OH (583 mg, 0.9 mmol, 3 equiv), Fmoc-Gln(Trt)-OH (549 mg, 0.9 mmol, 3 equiv), Fmoc-Tyr (tBu)-OH (413 mg, 0.9 mmol, 3 equiv), Fmoc-Ala-OH (280 mg, 0.9 mmol, 3 equiv), Fmoc-Pro-OH (304 mg, 0.9 mmol, 3 equiv), Fmoc-Glu (tBu)-OH (383 mg, 0.9 mmol, 3 equiv), Fmoc-D-Nle-OH (318 mg, 0.9 mmol, 3equiv), Fmoc-Trp (Boc)-OH (474 mg, 0.9 mmol, 3 equiv), F-moc Pro-OH, and Fmoc-Val-OH (305 mg, 0.9 mmol, 3 equiv) were coupled with the same manner. The resin and the side chain protected groups were removed from the peptide by shaking the peptidyl resin with a freshly prepared cocktail cleavage of TFA:TIS:water (95:2.5:2.5, *v/v/v*, 15 mL), for 3 h. The crude peptide was precipitated upon treatment with ice-cold ether followed by centrifugation, providing the targeted Fmoc-KK-11, which was subjected to RP-HPLC for purification (Retention time (R_t) = 31 min). The pure fraction was concentrated and subsequent freeze-drying to afford the pure powdered peptide. The purity of the peptide was confirmed by analytical RP-HPLC and the molecular weight by MALDI-TOF (m/z), [$C_{90}H_{118}N_{18}O_{18}$]: Calcd, 1738.9 Da; Found, 1740.5 Da $[M+2H]^+$. ^{1}H NMR (CD_3OD, 400 MHz, δ ppm): 7.26-7.92 (m, 19H, NH and NH_2 moieties), 6.62-7.20 (m, 22H, ArH), may be emerged, 3.60-4.54 (m, 10H, αCH, amino acids, Fmoc aliphatic H), 2.80-3.20 (m, 8H, βCH_2 Trp, Tyr, Phe and δCH_2 Arg), 1.45-2.38 (m, 35H, β,γCH_2 Arg, Pro, Gln, Glu, Nle; δCH_2 Pro, Nle, βCH_2, γCH Leu and βCH Val), 1.24 (d, J=7.2 Hz, 3H, βCH_3 Ala), 0.83-1.00 (m, 9H, $\gamma^1\gamma^2$ 2CH_3 Val, δ 2CH_3 Leu), 0.81 (d, J=6.4 Hz, 3H, δ 1CH_3 Leu), 0.73 (t, J=14.8 Hz, 3H, εCH_3, Nle).^{13}C NMR (CD_3OD, 100 MHz, δ ppm): 163.77-176.47 (15C, C=O), 157.44 (1C, Quanidine), 111.41-156.32 (33C, Ar), 51.26-66.78 (13C, αCH, amino acids and CH_2, Fmoc), 18.77-40.86 (24C, β,γ,δ amino acid carbons) 15.75 and 17.82 (2C, γ^1CH_3 and γ^2CH_3, Val), 14.35 (1C, βCH_3, Ala), 12.99 (1C, εCH_3, Nel).

2. VPWxEPAYQrFL (D-aa KK-11)

To yield peptide KK-11, the amino group of Fmoc-KK-11 was deprotected from Fmoc by 20% piperidine, followed by removing the resin and side chain protecting groups by shaking the peptidyl resin with a freshly prepared cocktail cleavage of TFA:TIS:water for 3 h. The crude deprotected peptide was precipitated using cold ether followed by centrifugation, providing the targeted crude peptide with free amine, which was subjected to RP-HPLC purification, R_t = 39 min. The pure fraction was concentrated and subsequent freeze-drying to afford the pure powdered peptide. The purity of the peptide was confirmed by analytical RP-HPLC and the molecular weight by MALDI-TOF (m/z), [$C_{75}H_{108}N_{18}O_{16}$]: Calcd, 1516.8 Da; Found, 1517.5 Da $[M+H]^+$. ^{1}H NMR (CD_3OD, 400 MHz, δ ppm): 7.51-8.19 (m, 20H, NH and NH_2 moieties), 6.62-7.29 (m, 14H, ArH), may be emerged, 3.94-4.48 (m, 10H, αCH, amino acids), 2.80-3.14 (m, 8H, βCH_2 Trp, Tyr, Phe and δCH_2 Arg), 1.46-2.37 (m, 35H, β,γCH_2 Arg, Pro, Gln, Glu, Nle; δCH_2 Pro, Nle, βCH_2, γCH Leu and βCH Val), 1.22 (d, J=7.2 Hz, 3H, βCH_3 Ala), 1.03 (d, J=7.2 Hz, 3H, γ^2CH_3 Val), 0.94 (d, J=7.6 Hz, 3H, γ^1CH_3 Val), 0.87 (d, J=6 Hz, 3H, δ 2CH_3 Leu), 0.82 (d, J=6 Hz, 3H, δ1CH_3 Leu), 0.72 (t, J=14.4 Hz, 3H, εCH_3, Nle). ^{13}C NMR (CD_3OD, 100 MHz, δ ppm): 167.64-176.17 (14C, C=O), 157.40 (1C, Quanidine), 108.99-156.25 (20C, Ar), 51.88-65.70 (12C, αCH, amino acids), 20.56-40.64 (24C, β,γ,δ amino acid carbons) 18.08 and 15.99 (2C, γ^1CH_3 and γ^2CH_3, Val), 14.27 (1C, βCH_3, Ala), 12.93 (1C, εCH_3, Nle).

a

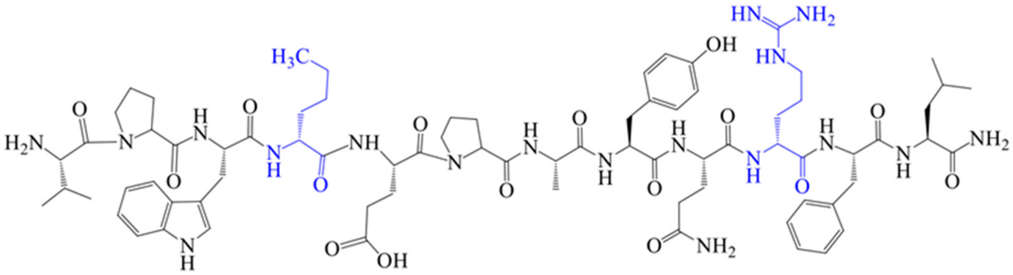
HN
NH2
OH
HN
H3C
O
H2N
N
H
N
O
N
H
NH
O
H
N
O
N
H
O
H
N
O
H
N
NH2
O
N
H
O
N
H
O
H
N
O
O
OH
O
NH2

b

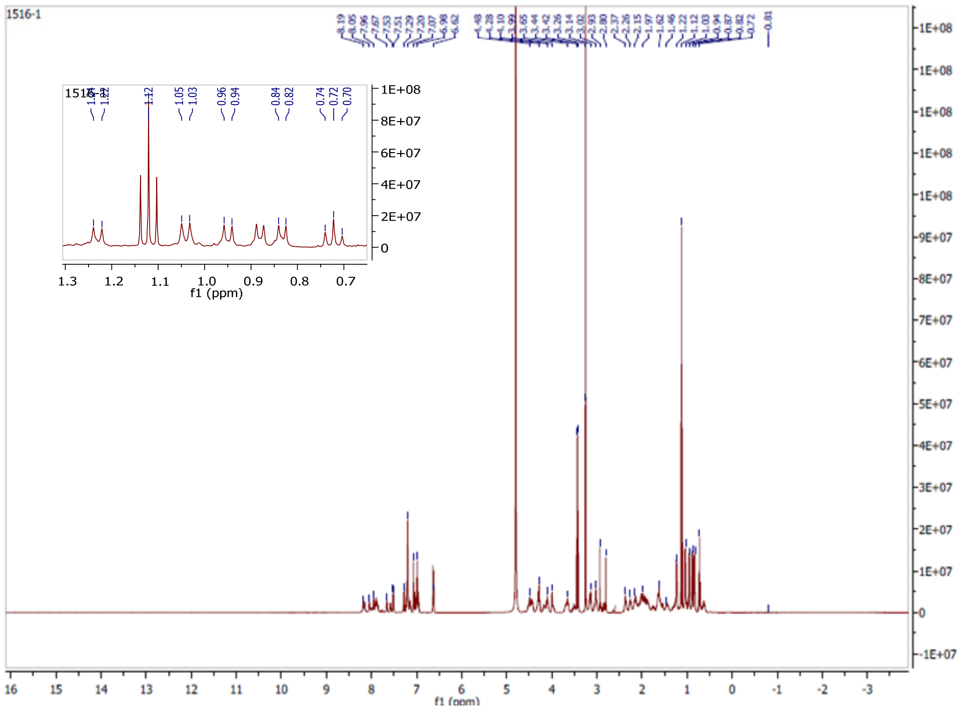
1516-1
f1 (ppm)

c

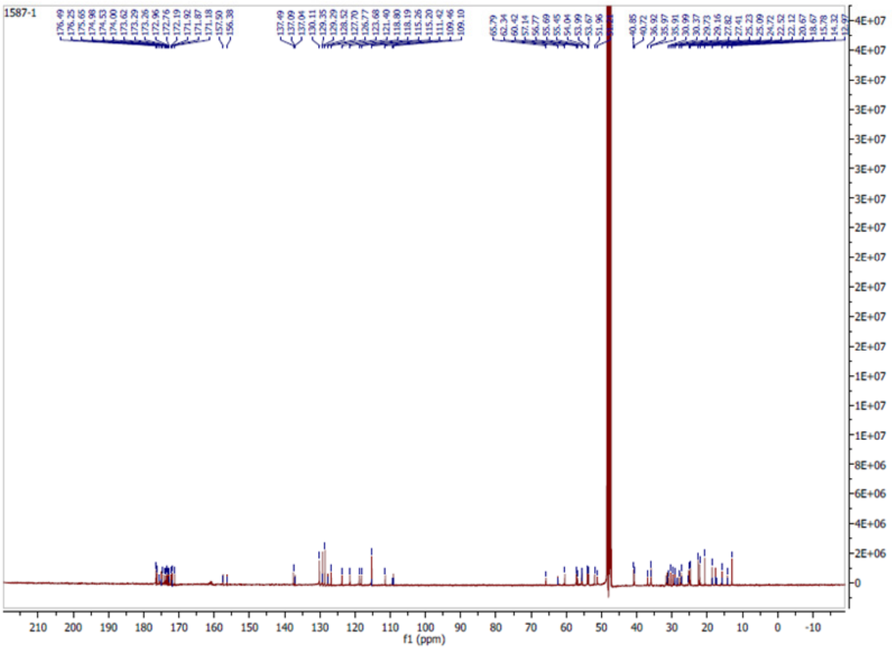

d

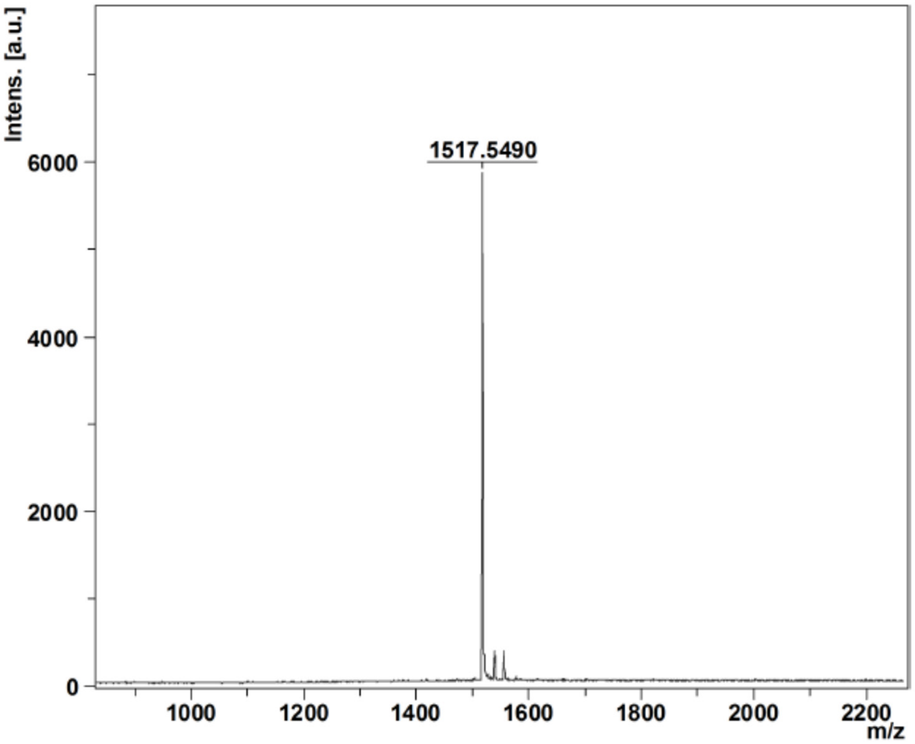

e

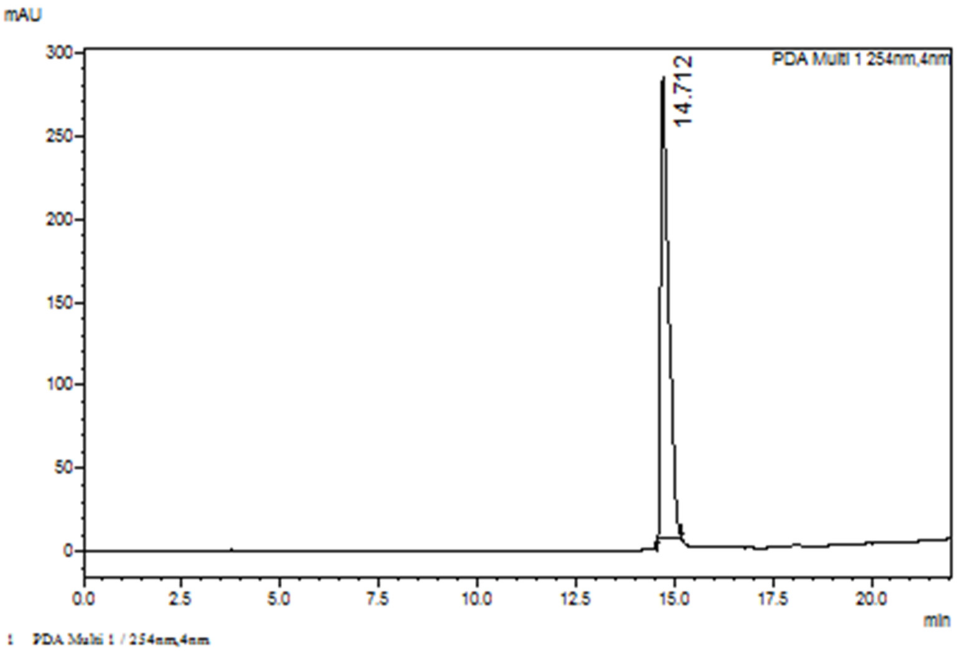

Figure S1. Peptide D-aa KK-11 (**a**) chemical structure, (**b**) H1 NMR, (**c**) C13 NMR, and (**d**) mass spectrum, and (**e**) analytical RP-HPLC chromatogram.

3. A_{β}VPWxEPAYQrFL (D-aa A_{β}-KK-11)

The same procedure was used for synthesis of KK-11 with an additional coupling using Fmoc-β-Ala-OH (280 mg, 0.9 mmol, 3 equiv) as a linker. The crude targeted peptide was subjected to RP-HPLC purification, R_t = 29 min. The pure fraction was concentrated and subsequent freeze-drying to afford pure powdered peptide A_{β}-KK-11. The purity of the peptide was confirmed by analytical RP-HPLC and the molecular weight by MALDI-TOF (m/z), [$C_{78}H_{113}N_{19}O_{17}$]: Calcd, 1587.9 Da; Found, 1588.4 Da $[M+H]^+$. ^{1}H NMR (CD_3OD, 400 MHz, δ ppm): 7.14-7.59 (m, 21H, NH and NH_2 moieties), 6.63-7.06 (m, 14H, ArH), may be emerged, 3.97-4.52 (m, 10H, αCH, amino acids), 2.80-3.12 (m, 8H, βCH_2 Trp, Tyr, Phe and δCH_2 Arg), 2.62 (t, 2H, J=16 Hz, αCH_2 βAla), 1.27-2.38 (m, 37H, β,γCH_2 Arg, Pro, Gln, Glu, Nle; δCH_2 Pro, Nle, βCH_2 Leu, βAla, γCH Leu and βCH Val), 1.23 (d, J=7.6 Hz, 3H, βCH_3 Ala), 0.88-0.94 (m, 9H, $\gamma^1\gamma^2$ 2CH_3 Val, δ 2CH_3 Leu), 0.83 (d, J=6.4 Hz, 3H, δ 1CH_3 Leu), 0.73 (t, J=14.4 Hz, 3H, εCH_3, Nle). ^{13}C NMR (CD_3OD, 100 MHz, δ ppm): 171.18-176.49 (15C, C=O), 157.50 (1C, Quanidine), 109.10-156.38 (20C, Ar), 51.96-65.79 (12C, αCH, amino acids), 20.67-40.85 (26 C, β,γ,δ amino acid carbons) 18.67 and 15.78 (2C, γ^1CH_3 and γ^2CH_3, Val), 14.32 (1C, βCH_3, Ala), 12.97 (1C, εCH_3, Nle).

a

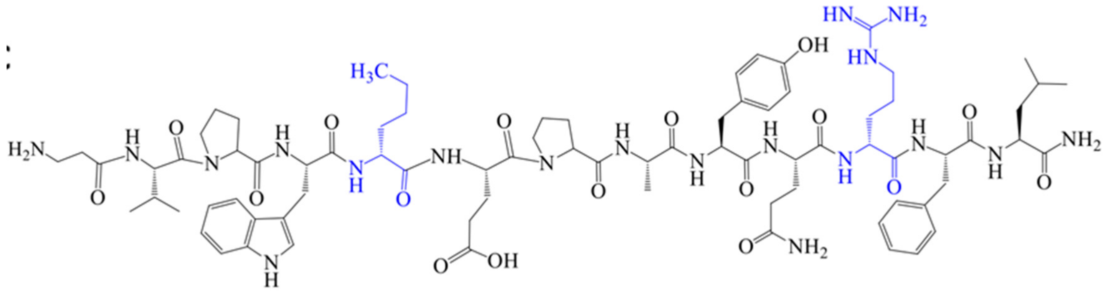

b

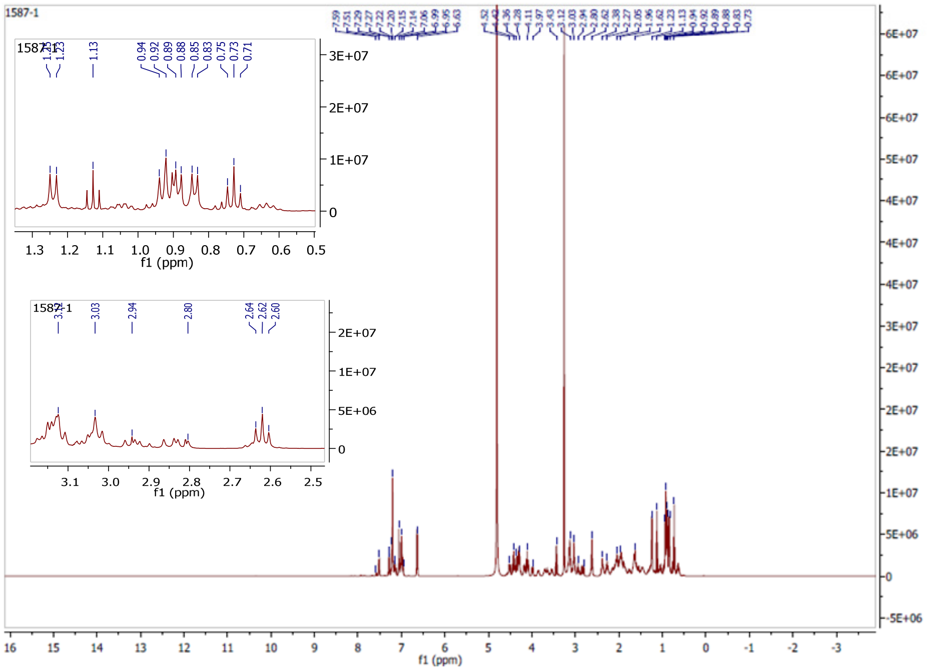

c

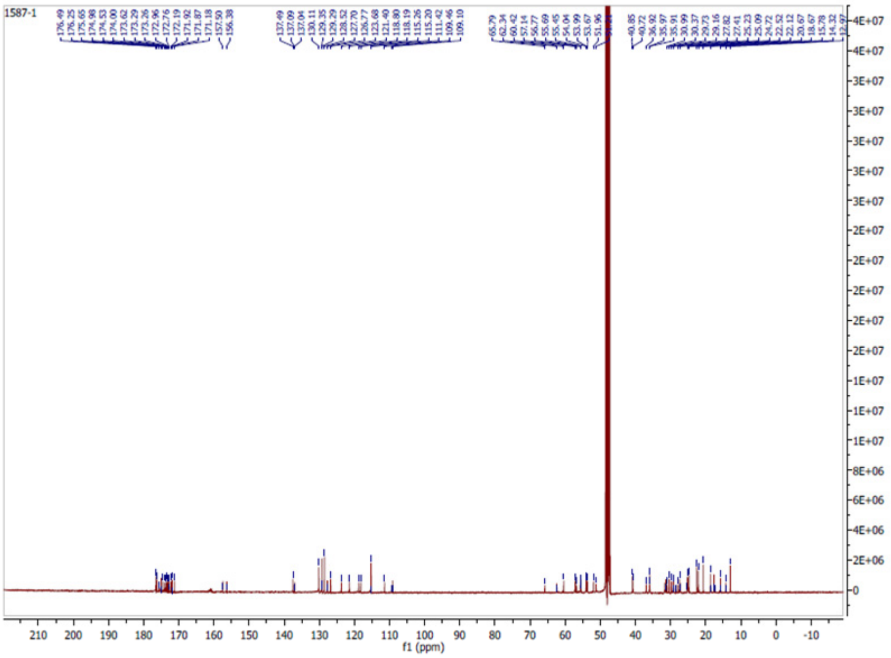

d

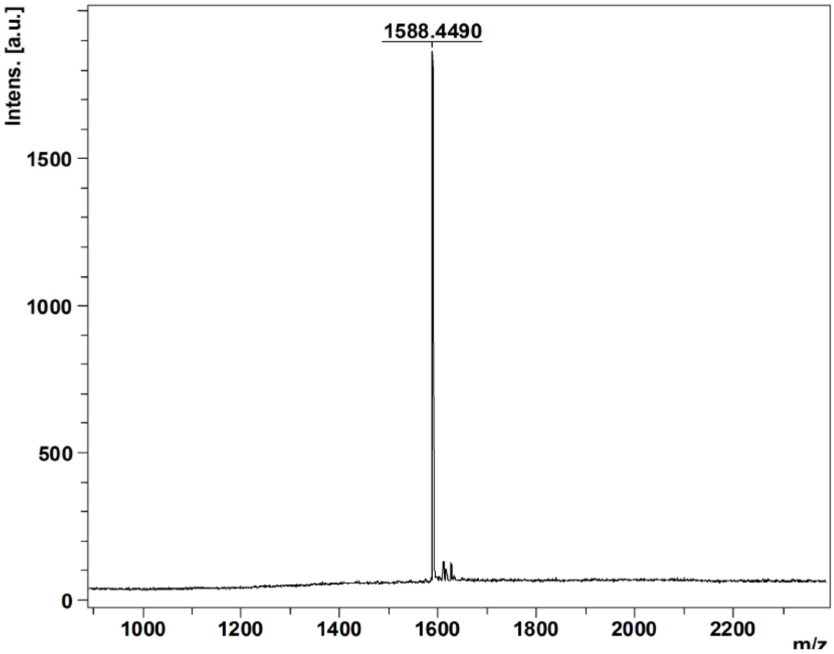

e

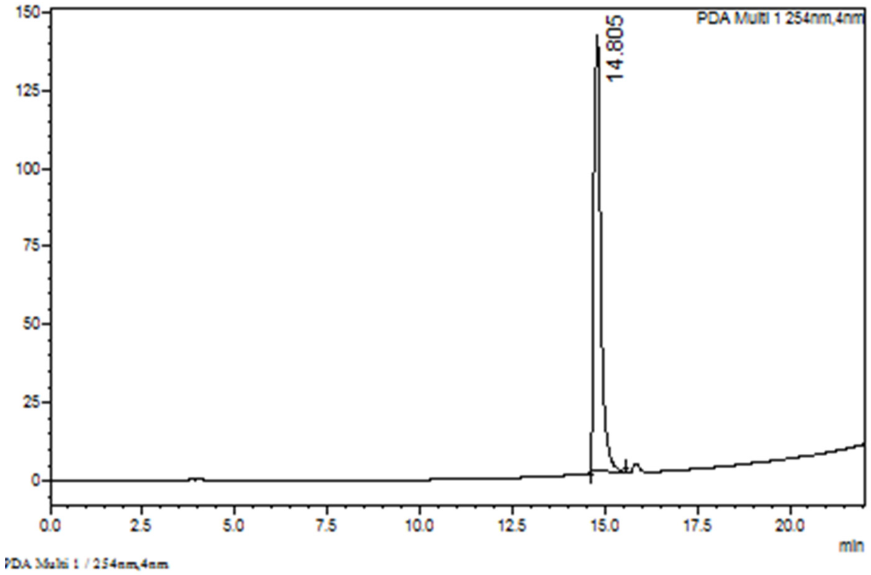

Figure S2. Peptide D-aa Aß-KK-11 (**a**) chemical structure, (**b**) H1 NMR, (**c**) C13 NMR, (**d**) mass spectrum, and (**e**) analytical RP-HPLC chromatogram.

a

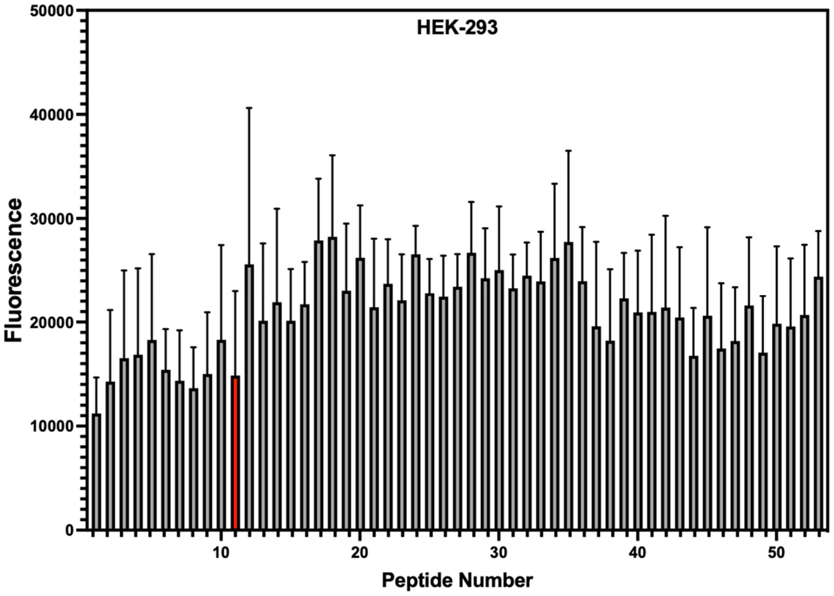

b

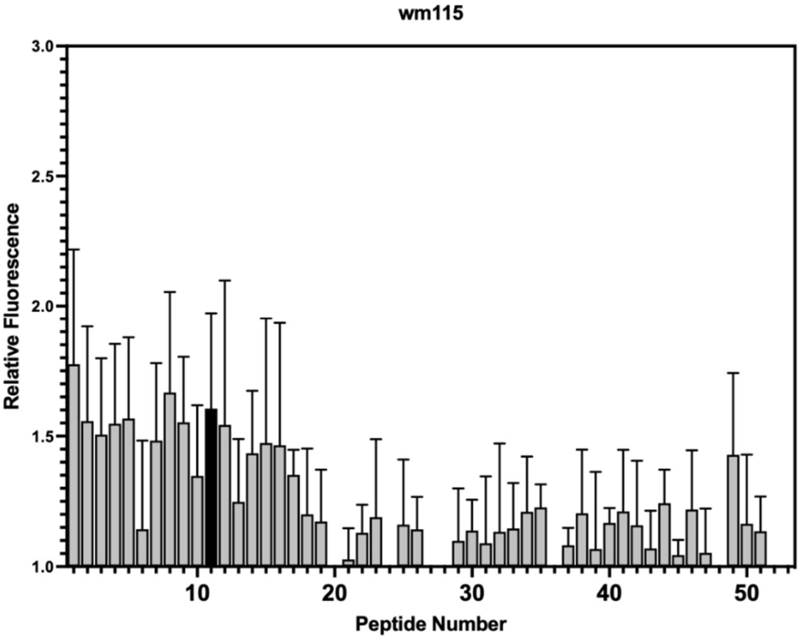

Figure S3. (**a**) Screening of peptide library (53 peptides in duplicates) for binding to HEK-293 cells; (**b**) Selective binding of peptides to human primary melanoma wm115 cells compared to HEK-293 cells. The binding fluorescence ratio of wm115 to HEK-293 represents the relative selectivity of peptide bindings with melanoma cells.

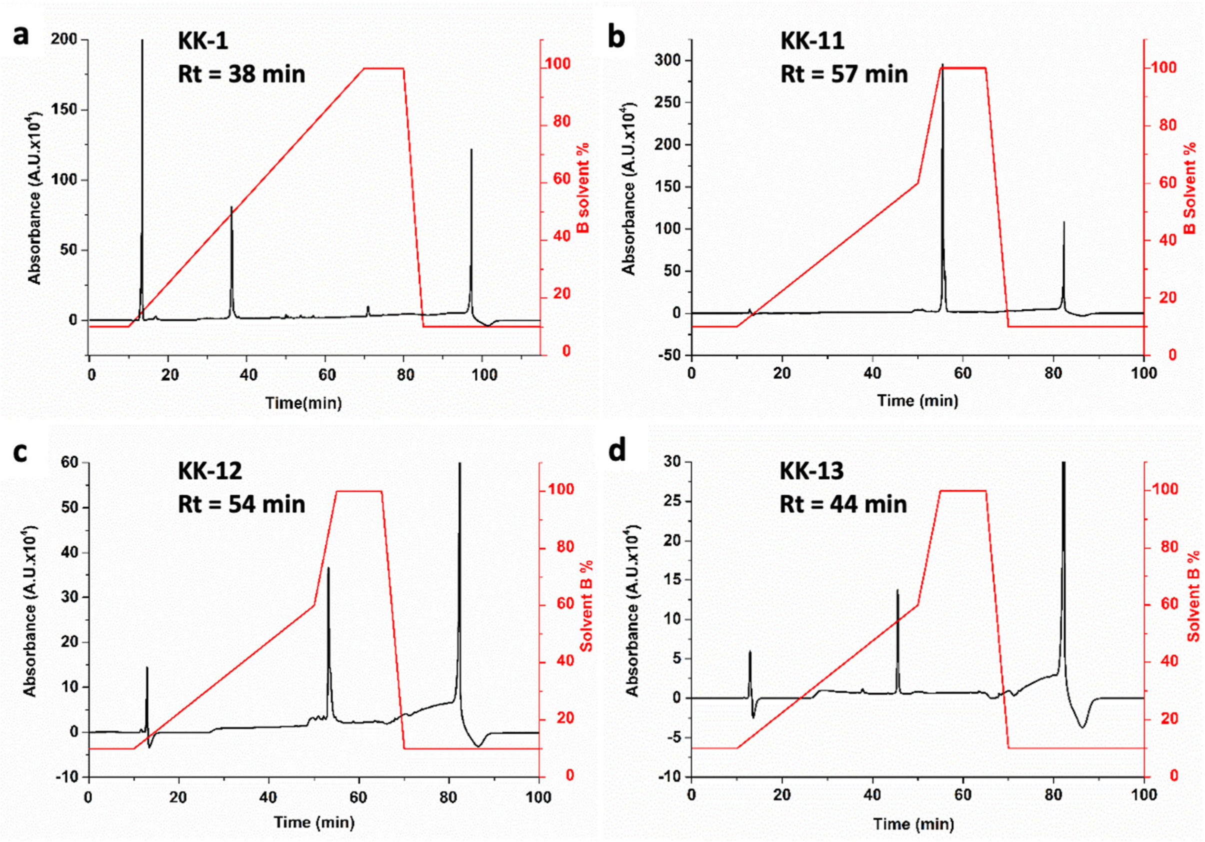

Figure S4. Analytical RP-HPLC chromatogram of FITC labelled peptides, (**a**) KK-1, (**b**) KK-11, (**c**) KK-12, and (**d**) KK-13. Gradient used on a Vydac C18 analytical column was as shown with 0.1%TFA in water as Sol A and 0.1%TFA in acetonitrile as solvent B, and the absorbance was monitored at 220 nm

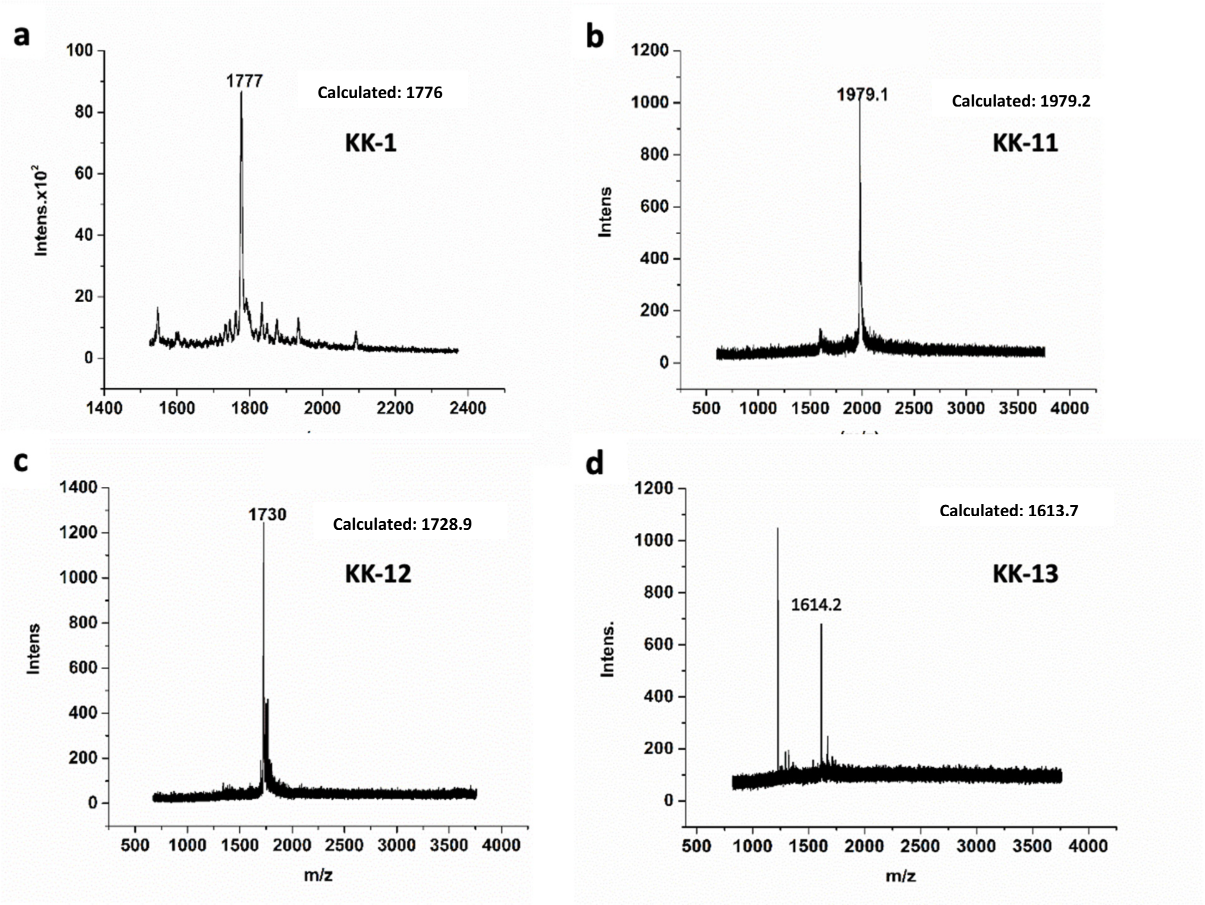

Figure S5. MALDI-TOF mass spectra for FITC labelled peptides.

a

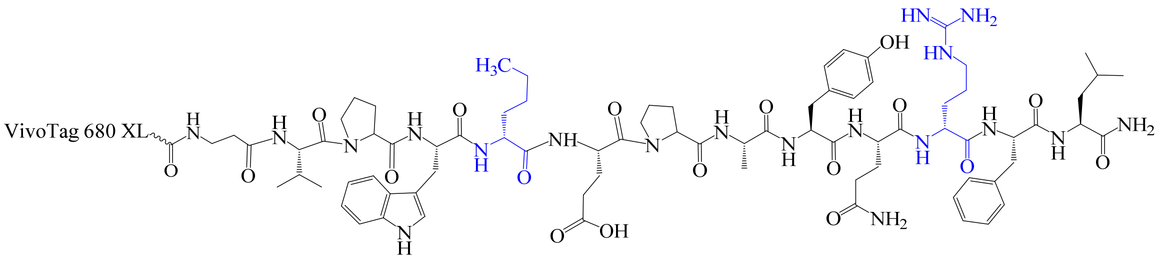

b

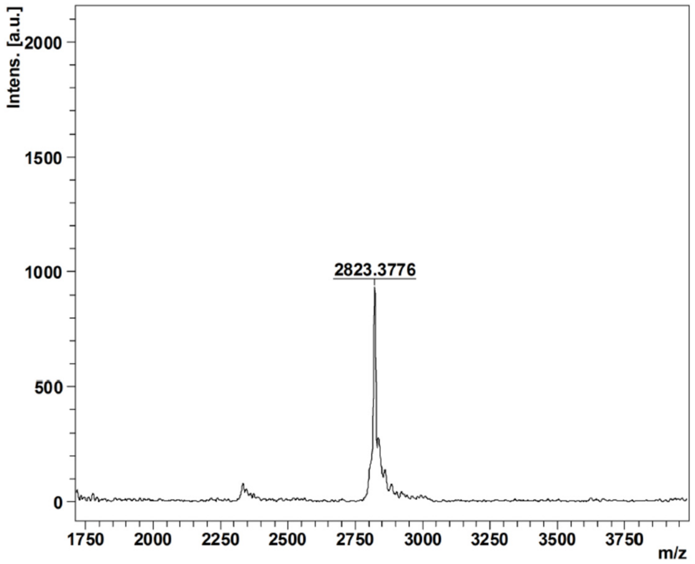

c

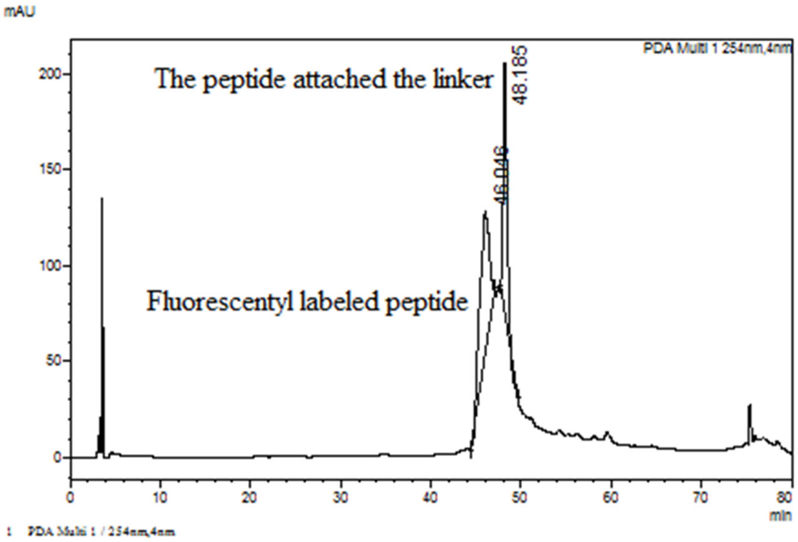

Figure S6. Peptide VivoTag 680 XL-Aß-KK-11 (**a**) chemical structure, (**b**) mass spectrum, and (**c**) analytical RP-HPLC chromatogram.

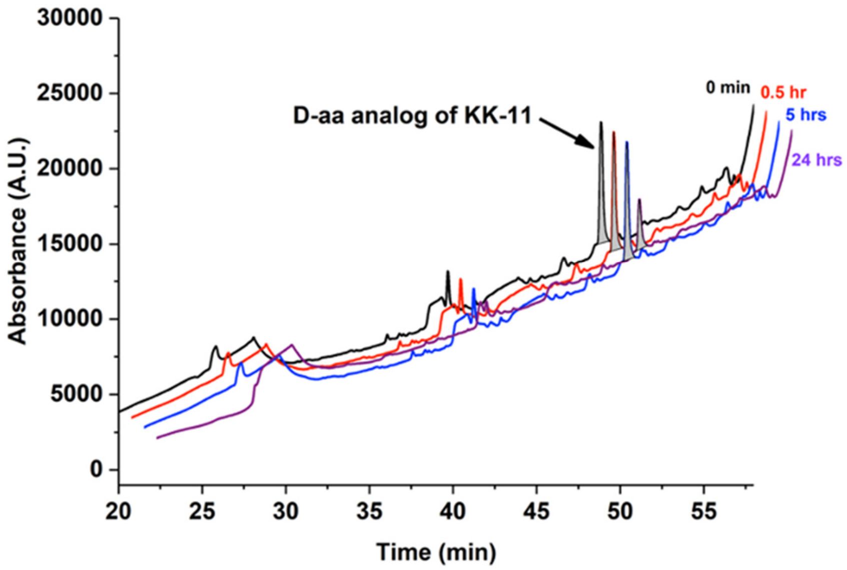

Figure S7. D-amino acid analogue of KK-11 exhibits increased serum stability. 100 µL of 1 mM of tested peptide dissolved in sterile water was added to pre-warmed DMEM medium containing 25% human serum. After vortexing, 100 µL of the final solution was added to 200 µL of methanol for RP-HPLC analysis after different incubation periods.
